# Establishment and phenotyping of disease model cells created by cell-resealing technique

**DOI:** 10.1038/s41598-017-15443-0

**Published:** 2017-11-09

**Authors:** Fumi Kano, Yoshiyuki Noguchi, Masayuki Murata

**Affiliations:** 1 Department of Life Sciences, Graduate School of Arts and Sciences, The University of Tokyo, 3-8-1 Komaba, Meguro-ku, Tokyo, 153-8902 Japan; 2 Cell Biology Center, Institute of Innovative Research, Tokyo Institute of Technology, 4259 Nagatsuta, Midori-ku, Yokohama, Kanagawa 226-8503 Japan; 3 Laboratory of Frontier Image Analysis, Graduate School of Arts and Sciences, The University of Tokyo, 3-8-1 Komaba, Meguro-ku, Tokyo, 153-8902 Japan

## Abstract

Cell-based assays are growing in importance for screening drugs and investigating their mechanisms of action. Most of the assays use so-called “normal” cell strain because it is difficult to produce cell lines in which the disease conditions are reproduced. In this study, we used a cell-resealing technique, which reversibly permeabilizes the plasma membrane, to develop diabetic (Db) model hepatocytes into which cytosol from diabetic mouse liver had been introduced. Db model hepatocytes showed several disease-specific phenotypes, namely disturbance of insulin-induced repression of gluconeogenic gene expression and glucose secretion. Quantitative image analysis and principal component analysis revealed that the ratio of phosphorylated Akt (pAkt) to Akt was the best index to describe the difference between wild-type and Db model hepatocytes. By performing image-based drug screening, we found pioglitazone, a PPARγ agonist, increased the pAkt/Akt ratio, which in turn ameliorated the insulin-induced transcriptional repression of the gluconeogenic gene phosphoenolpyruvate carboxykinase 1. The disease-specific model cells coupled with image-based quantitative analysis should be useful for drug development, enabling the reconstitution of disease conditions at the cellular level and the discovery of disease-specific markers.

## Introduction

Cell-based assays are increasing in importance for screening drugs and investigating their mechanisms of action. However, most of the assays use so-called “normal” cell strains, which do not reflect intracellular disease conditions. It is difficult to prepare cells that reflect pathological conditions from the tissues of patients for cell-based assays because primary differentiated cells do not proliferate sufficiently well to perform an entire series of experiments. In addition, these cells are normally a mixture of healthy cells and those in a pathological state, and such heterogeneity of cell samples makes commonly used biochemical analyses very difficult. Disease-specific cells that have been created by induced pluripotent stem (iPS) cell technology are quite promising for examining hereditary disease^1,2^, but might be unsuitable for lifestyle-related disease. Establishing a cell system in which the pathogenic conditions of a disease are reproduced should enable us to screen for drugs more effectively, elucidate their side effects, and determine their intracellular functional mechanisms under pathogenic conditions.

Understanding the mechanisms of cellular events under diabetic condition in pancreatic β cells, hepatocytes, and adipocytes has been the research focus of our group for years^3–7^. As part of the diabetes research, we previously established healthy and diabetic (disease) model cells from human cervical cancer-derived HeLa cells using the cell-resealing technique^3^.

Briefly, we prepared cytosol from the liver of a leptin receptor-deficient diabetic model mouse, a db/db mouse, and added it to semi-intact HeLa cells, whose plasma membranes had been permeabilized with streptococcal toxin, streptolysin O (SLO). The latter binds to cholesterol in the plasma membrane and oligomerizes to form pores of ~30 nm in diameter^8,9^. The SLO-mediated pores allow various molecules, such as proteins, nucleotides, and membrane-impermeable small molecules etc., to enter into cells. So semi-intact cell system enables the exchange of cytosol to the different one, which allowed us to reconstitute various intracellular phenomena such as morphological changes of the organelles during mitosis, the vesicular transport, and the organelle-specific targeting of proteins^10–14^. Then after the diabetic cytosol (Db liver cytosol) had been introduced into the cells, the plasma membrane was repaired by the addition of calcium ions to make the semi-intact cells intact again^15–20^. These cells are called “resealed cells”, and the resealed cells containing Db liver cytosol were used as “Db model cells”. By comparing the cellular phenotypes of Db model cells with those that contained wild-type liver cytosol (WT model cells) by various approaches, we could detect intracellular events that were specific to Db model cells under diabetic conditions. For example, p38 MAPK is activated in Db model cells, which results in a decrease in the amount of phosphatidylinositol-3-phosphate (PI3P) in early endosomes in Db model cells as compared with WT model cells^3^. Furthermore, we found that several endocytic pathways are perturbed in Db model cells: the retrograde transport of cholera toxin (Ctx) from endosomes to the Golgi apparatus is delayed in a p38 MAPK-dependent manner, whereas the degradation of the EGF receptor from endosomes to lysosomes is enhanced in a p38 MAPK-independent manner in Db model cells^3^. However, although we established a basic protocol for creating disease and healthy model cells and methods for analysing intracellular events under diabetic conditions, liver-specific phenotypes were not detected in the WT and Db cells derived from HeLa cells. It would be more useful to establish the resealed cells using hepatocyte-derived cells, because then liver-specific phenotypes, such as abnormalities in insulin-stimulated glucose metabolism that occur under diabetic conditions, could be evaluated directly.

In the study reported herein, we used rat hepatoma-derived H4IIEC3 cells to create diabetic model cells that contained Db cytosol (referred as to HDb cells) and control healthy model cells that contained WT cytosol (referred to as HWT cells). The HDb cells, but not HWT cells, showed perturbations in the expression of gluconeogenic genes and in the secretion of glucose into the medium, and therefore can be considered to reproduce insulin-resistant, pathological hepatocytic conditions. Furthermore, we performed image-based analysis of HWT and HDb cells to screen commercially available drugs. We propose that application of the disease-specific model cells created by using the cell-resealing technique, in combination with image-based analysis, could be a powerful platform for elucidating disease phenotypes at the cellular level to identify targets of therapy and also to screen drugs.

## Results

### Production of WT and Db model hepatoma-derived cells using the cell-resealing technique

**Figure 1:**
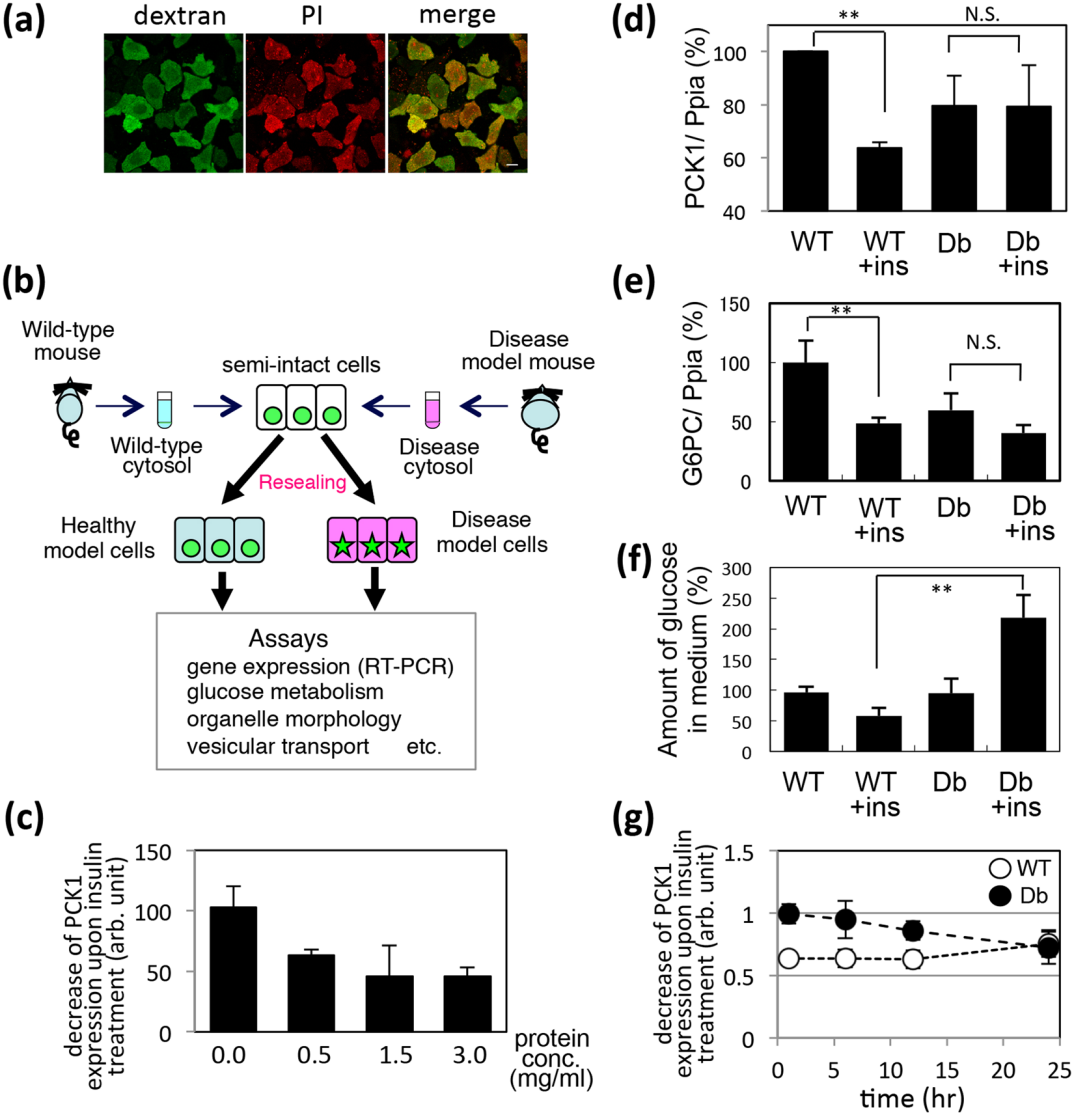
Establishment of healthy and diabetic model H4IIEC3 cells using the cell-resealing technique. (**a**) Resealed cells labeled with propidium iodide (PI, red) and dextran conjugated with fluorescein (dextran, green). Bar = 10 µm. (**b**) Scheme for the establishment and analysis of healthy or diabetic model H4IIEC3 cells. (**c**) Resealed H4IIEC3 cells that were introduced with WT liver cytosol at the protein concentration of 0.0, 0.5, 1.5, or 3.0 mg/ml were incubated with DMEM(-FBS) for 1 hours, and further with or without insulin for 1hr. The relative expressions of PCK1 upon insulin treatment to the one without insulin are analyzed and the means and standard deviations from four independent experiments are shown in the graph. (**d**) and (**e**) The relative expression of PCK1 (**d**) or G6PC (**e**) in the presence or absence of insulin treatment in HWT (WT) and HDb (Db) cells. The expression level of PCK1 or G6PC in WT cells was set to 100%. The means and standard deviations from three independent experiments are shown in the graph. **p = 0.08389 × 10^−6^ in (**d**) and **p = 0.0092 in (**e**). (**f**) HWT and HDb cells were incubated in glucose-free medium with (WT + ins or Db + ins) or without (WT or Db) insulin at 37 °C for 3 hrs. The amount of glucose in medium was quantified by glucose assay. The amount of glucose of WT cells was set to 100%. The means and standard deviations from three independent experiments are shown in the graph. **p = 0.00284. (**g**) HWT or HDb cells were incubated with DMEM(-FBS) for 1, 6, 12, and 24 hours, and further with or without insulin for 1hr. The expression level of PCK1 was analyzed by real-time PCR, and the relative expression of PCK1 upon insulin treatment to the one without insulin at each time points are analyzed in HWT cells (○) or in HDb cells (●) and the means and standard deviations from three independent experiments are shown in the graph.

First, based on the resealing protocol for HeLa cells described in Kano *et al*.^3^, we optimized the conditions for permeabilizing and resealing H4IIEC3 cells, a hepatoma cell line that is derived from rat liver, by varying the concentration of SLO and the incubation time and establishing the optimal resealing protocol for H4IIEC3 cells (see Methods). The resealed cells were observed as cells that were stained with PI (red) and fluorescein-dextran (green) (Fig. 1a), and the resealing efficiency, which was determined by morphometric analysis, was approximately 80–90%.

Using this procedure, we produced resealed H4IIEC3 cells, the cytosol of which was replaced with liver cytosol that had been prepared from WT or diabetic model db/db (Db) mice (Fig. 1b). After the introduction of WT or Db cytosol and resealing, the cells were incubated with medium at 37 °C and 5% CO_2_ for 1 hr to reduce the stress caused by the treatment with SLO. The resealed cells containing WT or Db cytosol are referred to HWT cells (hepatic wild-type model cells) or HDb cells (hepatic Db model cells) hereafter. To evaluate the replacement of the cytosol in the resealed cells, the leakage of cytosolic proteins from permeabilized cells was estimated by measuring the activity of lactate dehydrogenase (LDH), a cytosolic protein, in the supernatant after permeabilization. We found that 81.0 ± 7.8% of cytosolic LDH flowed out from the cells following SLO treatment, whereas 2.7 ± 0.2% leaked out of SLO-untreated intact cells. In addition, we confirmed that the inflow of added proteins was not affected by cytosolic condition, at least by WT or Db liver cytosol, since the amount of the exogenously-added GST protein was roughly similar between HWT and HDb cells (Supplementary Fig. S1a and S1b). Using the HWT and HDb cells, we observed the dissociation of EEA1 from endosomes, the depletion of phosphatidylinositol-3-phosphate in endosome, and the delay of the endosome-to-Golgi retrograde transport of Cholera toxin in HDb cells (Supplementary Fig. S1c–f), which were the same phenotype of the diabetic model HeLa cells that had been introduced with Db cytosol^3^.

### Perturbed expression of the gluconeogenic genes PCK1 and G6PC in HDb cells

Insulin resistance is the major phenotype for liver in type 2 diabetes mellitus. Consequently, we examined the response to insulin in HWT and HDb cells, by measuring the changes in expression of the gluconeogenic genes and the amount of glucose in the medium. First we confirmed whether the normal insulin-induced decrease in the transcription of PCK1, which is an enzyme in the gluconeogenic pathway, occurred in intact H4IIEC3 cells in a manner that was dependent on the concentration of cytosol. Semi-intact H4IIEC3 cells were incubated with WT cytosol at a concentration of 0.0, 0.5, 1.5, or 3.0 mg/ml at 37 °C for 30 min. After resealing and incubation with Dulbecco’s modified Eagle’s medium (DMEM) without fetal bovine serum (FBS) for 1 hr, the cells were incubated in the presence or absence of insulin for 1 hr. The amount of PCK1 mRNA was measured by quantitative real-time PCR (RT-PCR), and normalized against the amount of peptidylprolyl isomerase A (PPIA) mRNA. We observed a cytosol-dependent decrease in PCK1 mRNA upon insulin treatment (Fig. 1c). The absence of cytosol led to cell death, which might be one of the reasons for no response to insulin treatment under this condition. Cytosol with a protein concentration of >1.5 mg/ml appeared to be sufficient to induce the decrease in PCK1 mRNA upon insulin treatment. In accordance with our previous protocol for cell-based assays using resealed cells^3^, we used 3.0 mg/ml cytosol in the following experiments.

Next, we examined the response to insulin in HWT and HDb cells. HWT or HDb cells were incubated with DMEM (-FBS) for 1 hr and subsequently with insulin for 1 hr. The expression of PCK1 was measured by RT-PCR as described above. As shown in Fig. 1d, the amount of PCK1 mRNA decreased to ~60% of the original level upon insulin stimulation in HWT cells. In contrast, PCK1 expression was reduced subtly in HDb cells before insulin stimulation and was not downregulated significantly by insulin (Fig. 1d, Db and Db + ins). The same results were obtained by measuring the amount of mRNA that encoded G6PC (glucose-6-phosphatase, catalytic subunit), another enzyme in the gluconeogenic pathway (Fig. 1e).

We also performed glucose assays to measure the amount of glucose in the medium. Insulin-stimulated reduction of glucose in the medium was observed for HWT cells, but the glucose level remained high for HDb cells after stimulation with insulin (Fig. 1f). Thus, glucose metabolism was perturbed in HDb cells, compared to HWT cells.

We tested how long the effect of introduced cytosol was maintained functionally in HWT and HDb cells. HWT or HDb cells were incubated for 1, 6, 12, or 24 hrs and then for a further hour with insulin. The expression of PCK1 was measured by RT-PCR and the decrease in PCK1 expression is shown in Fig. 1g. Given that the effect of the introduced cytosol unexpectedly lasted for 12 hrs after resealing, the assays in this study were performed within at least 12 hrs after resealing.

To test whether the perturbed glucose metabolism was also reproduced in cells that contained liver cytosol prepared from a different diabetic mouse model, we used liver cytosol prepared from a mouse with high-fat diet-induced obesity (HF)^21^. The liver cytosol of an Apolipoprotein E (ApoE)-deficient mouse, a model for atherosclerosis^22^, was also used as another disease-related cytosol. The expression of PCK1 and G6PC in the presence or absence of insulin was measured by RT-PCR in resealed cells that contained HF or ApoE cytosol. Interestingly, the insulin-induced decrease in PCK1 and G6PC expression was disturbed in HF cells and the cells were less sensitive to insulin than HWT cells (Supplementary Fig. S2a and S2b, HF). In contrast, no significant difference could be seen between HWT and ApoE cells (Supplementary Fig. S2a and S2b, ApoE). Thus, the impaired expression of PCK1 and G6PC might be a common phenotype when cytosol from diabetic model mice is introduced into cells.

It is reported that the expression of sterol response element binding protein 1c (SREBP1c), a transcription factor regulating the lipogenic gene expression, was elevated in diabetic model ob/ob mouse^23^ and db/db mouse^24^, and upon insulin treatment. However, whereas insulin-mediated elevation of SREBP1c expression was confirmed in intact H4IIEC3 cells (Supplementary Fig. S2c), we found no increase in SREBP1c expression in HWT and HDb cells and upon insulin stimulation (Supplementary Fig. S2d).

### Perturbation of Akt signaling in HDb cells

**Figure 2:**
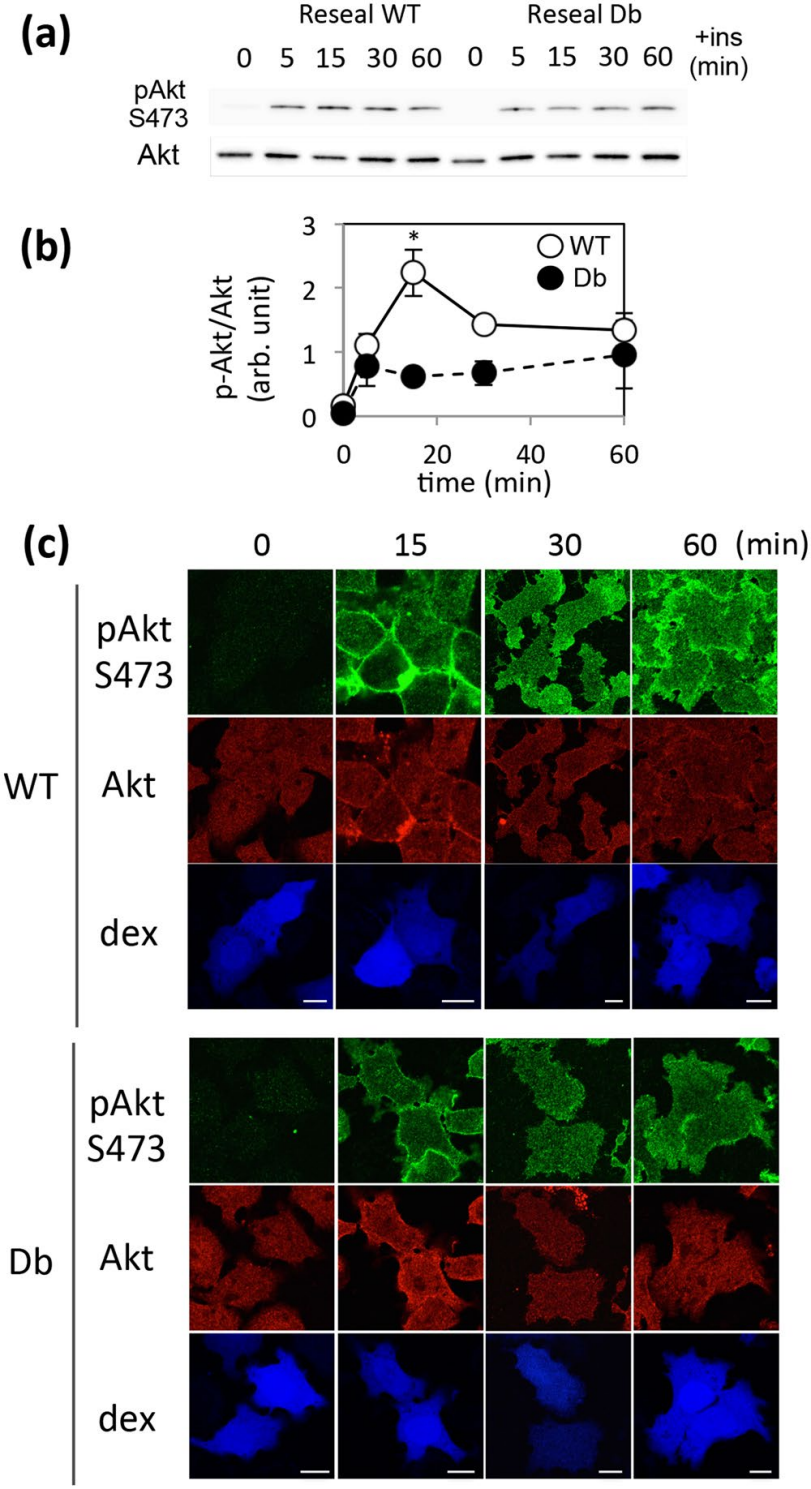
Disturbance of Akt phosphorylation in HDb cells. (**a**) After serum starvation, H4IIEC3 cells were permeabilized and introduced with WT or Db liver cytosol. After resealing and the incubation with DMEM(-FBS), the cells were stimulated with insulin for the indicated times, lysed, and subjected to western blotting using antibodies against pAktS473 and Akt. (**b**) The bands for pAktS473 and Akt in WT (○) and Db (●) cells were quantified, and the mean ratios of pAktS473 to Akt and the s.e.m. from three independent experiments are shown in the graph. *p = 0.03664. (**c**) HWT (WT) or HDb (Db) model cells were treated with insulin for the indicated times and subjected to immunofluorescence using anti- phosphorylated Akt at S473 (pAktS473) and anti-Akt antibody, which recognizes Akt1 and some Akt2 (30% sensitivity compared to Akt1). “dex” indicates the fluorescently-labeled dextran introduced into cells with cytosol as a marker of resealed cells. Bar = 10 µm.

Transcription of PCK1 and G6PC is regulated via PI3K-Akt signal transduction. We focused on Akt, one of the key kinases in the insulin signaling pathway^25,26^, and compared the difference in the level of Akt activation in HWT cells and HDb cells. HWT or HDb cells were incubated with DMEM without serum for 1 hr, and then treated with insulin for the indicated times. The cells were subjected to WB and immunofluorescence analyses. Interestingly, WB analysis revealed that the amount of phosphorylated Akt at Ser473 was decreased in HDb cells 15 min and 30 min after insulin treatment whereas the amount of total Akt was unaffected (Fig. 2a and b), and the difference was statistically significant at 15 min (Fig. 2b). Immunofluorescence analysis demonstrated that the fluorescence signal for pAktS473 appeared to be diminished in HDb cells during treatment with insulin for 15–60 min as compared to that observed in HWT cells (Fig. 2c). Although the time point at which the reduced phosphorylation of Akt in Db cells was detected by either WB and immunofluorescence analyses differed, both analyses demonstrated inhibition of Akt phosphorylation in HDb cells (these differences are discussed in the Discussion).

### Phenotyping of HWT and HDb cells at single cell level by immunofluorescence using quantitative image analysis

Resealed cells are easily discernible by fluorescence microscopy because fluorescent markers can be introduced through the cytosol. Consequently, it was possible to extract data from immunofluorescence images of a single HWT or HDb cell. This enabled us to compare quantitatively the differences between single HWT and HDb cells and to process image data statistically after we had obtained a large quantity of data.

**Figure 3:**
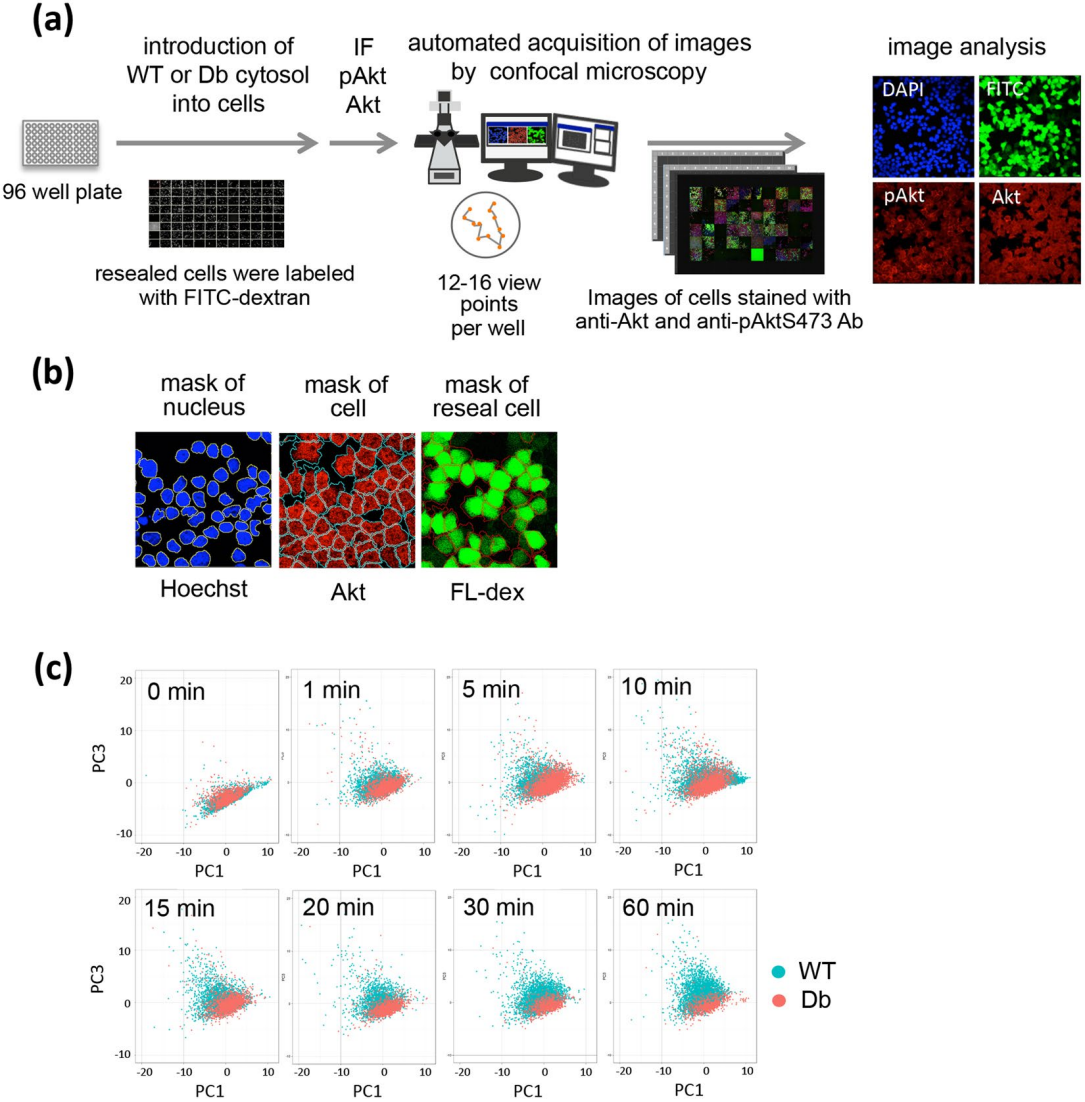
Analysis of immunofluorescence images of HWT and HDb cells stained with antibodies against pAktS473 and Akt. (**a**) Schematic representation of the automated image acquisition system and the image analysis. (**b**) The mask of nucleus, cell, and resealed cells were determined by the binary images of Hoechst, Akt, fluorescein-dextran (FL-dex) staining. (**c**) HWT or HDb cells were treated with insulin for the indicated times and subjected to immunofluorescence using antibodies against pAktS473 and Akt. The feature quantities obtained from the images were subjected to principal component analysis. The individual HWT (blue) and HDb (pink) cells were plotted in a PC1-PC3 graph.

To obtain and analyze a large quantity of immunofluorescence image data from single cells, we built an automated high-throughput fluorescence-based cell imaging system that enabled quantitative image analysis using NIS-Elements (Nikon). A schematic representation of the high-throughput image acquisition and analysis system is shown in Fig. 3a. HWT or HDb cells on 96-well plates were treated with insulin for 0, 1, 5, 10, 15, 20, 30, or 60 min at 37 °C. The cells were fixed and stained with anti-pAktS473 antibody, anti-Akt antibody, and Hoechst 33342 (a nuclear stain), and observed automatically by confocal microscopy. We observed that the fluorescence signal of pAktS473 and Akt was significantly reduced in response to pretreatment of the antibodies with immunized peptide (Supplementary Fig. S3), confirming that the staining was specific to each antibody. Z-stack images of 12 to 16 points per well were obtained using the automated image acquisition system: we obtained ~100 images (12–16 points × 8 Z-stack images) per well in this experiment. The areas of the nucleus, cell, and resealed cells could be detected using NIS-elements software (Fig. 3b). In addition, we obtained data only from resealed cells, which were labeled with fluorescein-dextran. We extracted a variety of quantitative data, namely the mean, sum, maximum, minimum, and standard deviation of fluorescence intensity for pAkt, Akt, Hoechst 33342, and fluorescein in the area of the whole cell, nucleus, and cytosol at single cell level. We call these quantitative data “feature quantities”, and they are shown in Supplementary Table S1.

To find out which feature quantities should be used to distinguish between HWT and HDb cells, we performed principal component analysis (PCA) using these feature quantities. The loadings are shown in Supplementary Table S2. Given that PC2, which relates to the fluorescence intensity of fluorescein, did not separate HWT and HDb cells, we used PC1 and PC3 for this analysis. When plotted in a PC1–PC3 graph, individual cells of either the HWT or HDb cell group localized to a merged area in the initial state (time point, 0 min), but were distributed separately as the time of incubation with insulin increased (Fig. 3c). The position of individual HWT cells moved to an upper area on the PC3 axis upon insulin stimulation, whereas that of the HDb cells remained unchanged, which indicated that the ratio of pAktS473 to Akt was increased in HWT cells upon insulin treatment but not in HDb cells. Thus, we chose the ratio of the amount of pAktS473 to Akt as an index to distinguish between HWT and HDb cells.

### Screening of small-chemical compounds that modulated the ratio of pAkt to Akt in HWT and HDb cells

We applied the above quantitative image-based analytical system on a trial basis to screen for compounds that could change this ratio, indicating amelioration or pejoration of insulin resistance in HDb or HWT cells. For the screening, we used a library of small chemical compounds that contained 90 drugs and was developed by Pfizer (LOPAC Pfizer), in addition to seven drugs, artificial sweeteners, and hormones used in the treatment of diabetes mellitus (pioglitazone, metformin, glibenclamide, nateglinide, [D-Lys3]-GHRP-6, sucralose, saccharin).

Practically, HWT or HDb cells on 96-well plates were treated with small chemical compounds from LOPAC-Pfizer at 1 µM or 10 µM or the seven additional drugs (the working concentrations are described in Methods) for 60 min and then 100 nM insulin was added for 15 (10 µM) or 60 min (1 and 10 µM). The cells were further subjected to immunofluorescence using anti-pAktS473 and anti-Akt antibodies as described above. We confirmed the lack of background fluorescence by the drugs at the wavelength used for imaging pAktS473 (546 nm) and Akt (647 nm), although E9, H9, and A11 caused strong or weak fluorescence at 488 nm (Supplementary Fig. S4). As such, the fluorescence signal specifically from resealed cells exposed to these agents might be lower in accuracy. In addition, the Z’-factor, which is a value for assessing assay quality, was 0.5013, suggesting that this assay was well controlled.

**Figure 4:**
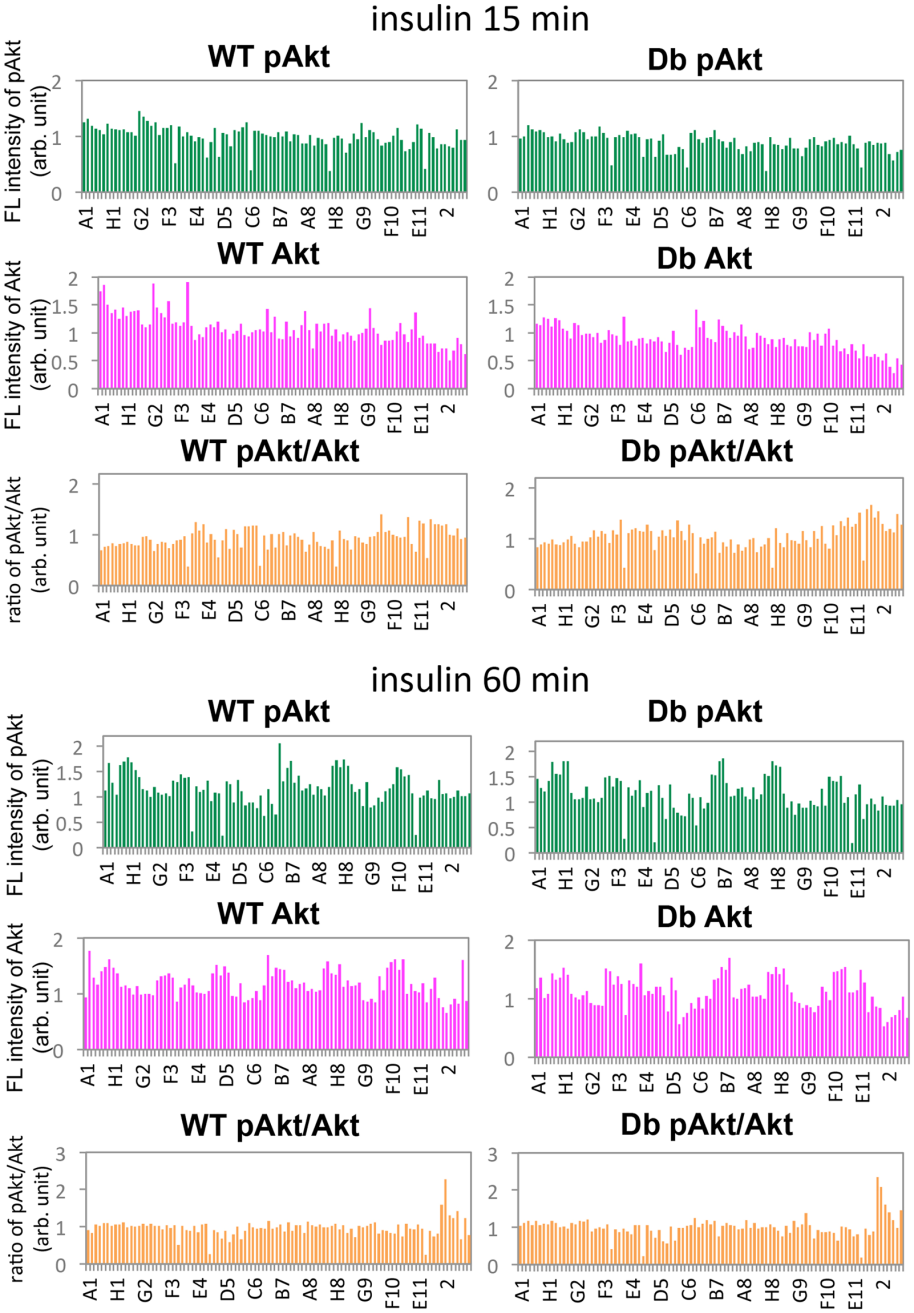
Quantification of the mean fluorescence intensity of pAktS473 and Akt, and the ratio of pAktS473 fluorescence to Akt fluorescence in HWT and HDb cells treated with a library of small chemical compounds. Serum-starved H4IIEC3 cells that had been grown on 96-well plates were permeabilized with SLO, and incubated with WT or Db liver cytosol that contained dextran conjugated with fluorescein. After resealing and subsequent incubation with DMEM(-FBS) for 1 hr in the presence of small chemical compounds, the cells were treated with insulin for 15 or 60 min and subjected to immunofluorescence using anti-pAktS473 and anti-Akt antibodies. The images were obtained by using the automated image acquisition system. The mean fluorescence intensities of pAktS473 and Akt, and the mean ratio of pAktS473 fluorescence to Akt fluorescence are shown in the graph.

After acquiring and analyzing the images and obtaining the ratio of the mean fluorescence of pAktS473 to the mean fluorescence of Akt, we found no significant increase or decrease of the mean fluorescence intensity of either pAktS473 and Akt or the ratio of pAkt to Akt in drug-treated HWT and HDb cells at a concentration of 1 µM (Supplementary Fig. S5a). From the screening result at the concentration of 10 µM, we identified three drugs that lowered the ratio, and two drugs that elevated it. Avasimibe (Avs; an ACAT inhibitor), Crizotinib (Crz; a c-Met and ALK inhibitor), PD-184161 (a MEK inhibitor), PD-184352 (a MKK1 inhibitor), and PF-431396 (PF; a PYK2 and FAK inhibitor) were found to suppress the phosphorylation of Akt after treatment with insulin for 15 min (Fig. 4, insulin 15 min). However, Akt phosphorylation remained inhibited after 60 min in cells that were treated with Avs, Crz, or PF (Fig. 4, insulin 60 min), but was restored in those exposed to PK-184151 or PK-184352. Pioglitazone (Pio; a PPARγ agonist, an anti-diabetic drug) and metformin (Met; an anti-diabetic drug) increased the ratio of pAktS473 to Akt at 15 and 60 min after insulin addition in both HWT and HDb cells (Fig. 4). Compared with Met, which increased the ratio by 2.275 in HWT cells and 2.077 in HDb cells, Pio appeared to affect HDb cells more specifically (an increase of 1.596 in HWT cells and 2.347 in HDb cells). Interestingly, the fluorescence intensity of pAkt was not increased by Pio or Met but rather the fluorescence of total Akt decreased, which led to an increased ratio of pAktS473 to Akt. In addition, we evaluated the effect of Avs, Crz, PF, and Pio at 5 µM and Met at 1 mM on the pAkt/Akt ratio, and found a modest decrease in the latter in response to Crz or PF and an increase in response to Met (Supplementary Fig. S5b).

### Evaluating the effect of the screened drugs on Akt phosphorylation and the insulin-induced transcriptional repression of gluconeogenic genes in intact H4IIEC3 cells

**Figure 5:**
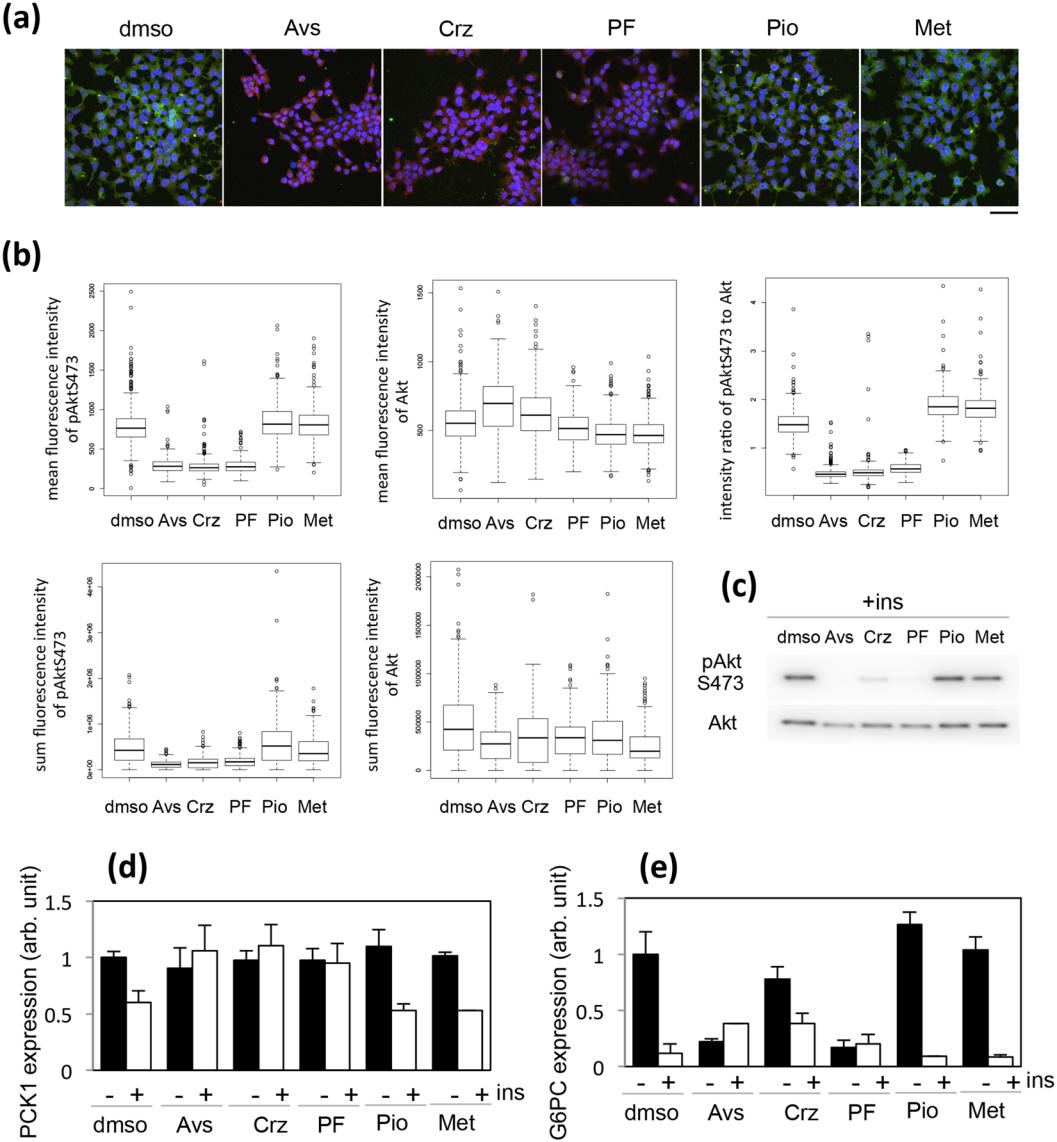
Effect of five drugs identified by screening a drug library on the phosphorylation and amount of Akt, and the insulin-mediated transcriptional regulation of PCK1 and G6PC. (**a**) Serum-starved H4IIEC3 cells were treated with DMSO, 10 µM Avs, 10 µM Crz, 10 µM PF, 10 µM Pio, or 2 mM Met for 1 hr, and then with 100 nM insulin for a further 1 hr. The cells were stained with antibodies against pAktS473 (green) and Akt (red), and Hoechst 33342 (blue). Bar = 50 µm. (**b**) The immunofluorescence images obtained in (**a**) were examined by image-based analysis. The mean or sum fluorescence intensities of pAktS473 and Akt and the ratio of pAkt fluorescence to Akt fluorescence are shown in the box plot. (**c**) The cells were treated as described in (**a**), lysed, and subjected to western blotting using antibodies against pAktS473 and Akt. (**d**) and (**e**) The cells were treated with DMSO and each of the five inhibitors as described in (**a**), and then in the presence or absence of 100 nM insulin for a further 1 hr. The relative expression levels of PCK1 (**d**) and G6PC (**e**) were obtained by RT-PCR. The expression levels of PCK1 and G6PC in DMSO-treated cells were set to 100%. The means and standard deviations from three independent experiments are shown in the graph.

The effect of the five compounds identified above was evaluated in intact H4IIEC3 cells by using the above-mentioned quantitative image-based analytical system and anti-pAktS374 or anti-Akt antibody (Fig. 5a and Supplementary Fig. S6). The results were consistent with the ones obtained during screening. Avs, Crz, and PF significantly decreased the fluorescence signal of pAkt without changing the total amount of Akt in intact cells (Fig. 5b, Avs, Crz, and PF), rather the mean fluorescence of Akt was upregulated by Avs and Crz, which might be caused by a shrinking of the cell area because the sum intensity of Akt was not changed in Avs- or Crz-treated cells (Fig. 5b, Avs and Crs). In contrast, Pio and Met increased the ratio of pAkt to Akt (Fig. 5b, intensity ratio of pAktS473 to Akt, Pio and Met). This could be attributed to a decreased fluorescence signal for total Akt (Fig. 5b, mean fluorescence intensity of Akt). The phosphorylation state of Akt was also examined by WB. Whereas the inhibition of phosphorylation of Akt by Avs, Crz, and PF was confirmed by WB, no substantial decrease in the amount of Akt was observed upon treatment with Pio or Met (Fig. 5c, see Discussion).

Next, we examined the effects of the five compounds on transcriptional repression of the PCK1 and G6PC genes in intact H4IIEC3 cells after insulin stimulation. In the presence of Avs, Crz, and PF, intact H4IIEC3 cells showed an insulin-resistant phenotype with respect to the transcriptional regulation of PCK1 (Fig. 5d), whereas G6PC transcription was downregulated even before insulin treatment (Fig. 5e). These results suggested that the inhibition of Akt might affect PCK1 and G6PC expression differently: the insulin-induced decrease in PCK1 expression might be dependent on Akt whereas G6PC expression might require Akt but be independent of insulin. In contrast, Pio and Met did not lead to a significant perturbation in the transcriptional regulation of PCK1 and G6PC in intact H4IIEC3 cells (Fig. 5d and e).

### Quantitative image-based analysis and effect of insulin on gluconeogenic gene expression of pioglitazone-treated HWT and HDb cells

**Figure 6:**
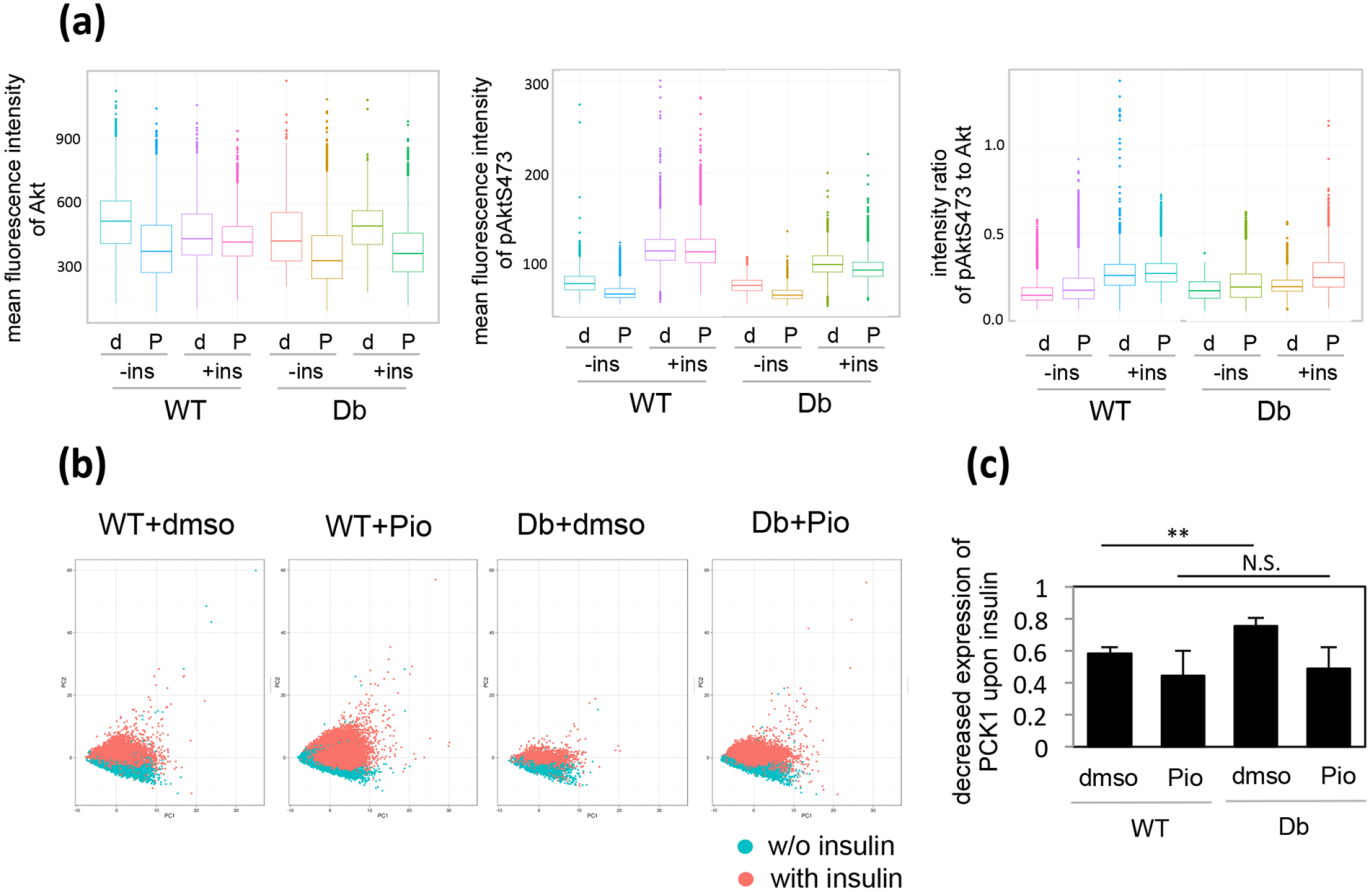
Pioglitazone restored the decreased ratio of pAktS473 to Akt and the perturbed expression of PCK1 in HDb cells. (**a**) HWT (WT) or HDb (Db) cells were treated with DMSO (**d**) or 10 µM Pio (P) for 1 hr, and then in the presence (+ins) or absence (−ins) of insulin for a further 1 hr. The cells were stained with antibodies against pAktS473 and Akt. The mean fluorescence intensities of pAktS473 and Akt and the intensity ratio of pAkt fluorescence to Akt fluorescence are shown in the box plot. (**b**) PCA analysis of the fluorescence images of pAktS473 and Akt in HWT and HDb cells treated with DMSO (WT + dmso or Db + dmso) or Pio (WT + Pio or Db + Pio) obtained as described in (**a**). The individual cells treated with (pink) or without (blue) insulin were plotted in the graph. (**c**) HWT and HDb cells were treated as described in (**a**), and the relative expression of PCK1 was examined by RT-PCR. The relative expression of PCK1 upon insulin treatment to the one without insulin was analyzed. The means and standard deviations from three independent experiments are shown in the graph. **p = 0.008868785.

Given that we detected a more specific effect of Pio on the pAkt/Akt ratio in HDb cells than in HWT cells during screening, the precise effect of Pio was evaluated in HWT and HDb cells by using the above-mentioned quantitative image-based analytical system. The image data for HWT and HDb cells treated with dimethyl sulfoxide (DMSO; as a control) or Pio in the presence or absence of insulin were analyzed and the results are shown as a box plot in Fig. 6a. Treatment with Pio reduced the total amount of Akt in either HWT or HDb cells regardless of insulin treatment (Fig. 6a, WT and Db). In contrast, the level of pAktS473 was decreased but only by a relatively small amount compared to the decrease in Akt (Fig. 6a, mean fluorescence intensity of pAktS473). Furthermore, whereas the pAkt/Akt ratio did not increase upon insulin stimulation in DMSO-treated HDb cells, Pio restored the ability of insulin to increase the ratio to the same level as that seen in DMSO-treated HWT cells (Fig. 6a, intensity ratio of pAktS473 to Akt). The pAkt/Akt ratio was elevated more markedly by Pio in insulin-treated HDb cells than in insulin-treated HWT cells.

The image data were subjected to PCA analysis as described above. The loadings are shown in Supplementary Table S3. Whereas the distribution of DMSO-treated HDb cells in the presence and absence of insulin overlapped (Fig. 6b, Db + dmso), after treatment with Pio, the insulin-treated HDb cells moved to the same location as HWT cells that had been treated with insulin, meaning that the pAkt/Akt ratio was increased (Fig. 6b, Db + Pio). Pio also increased the pAkt/Akt ratio in HWT cells as shown by the movement upwards of the cell area for HWT + Pio as compared with HWT + DMSO (Fig. 6b, WT + dmso and WT + Pio). We also found that Pio treatment restored the insulin-dependent transcriptional regulation of the PCK1 gene in HDb cells to the same level as that in HWT cells (Fig. 6c).

Therefore, using our quantitative image-based analytical system and quantitative RT-PCR method, we confirmed that Pio specifically and significantly improved the phenotype of HDb (disease) cells to that of HWT (healthy) cells by increasing the pAkt/Akt ratio.

## Discussion

The first aim of this study was to create hepatic diabetic model cells, in which insulin resistance was replicated, by using our cell-resealing technique with a liver-derived cell strain. We have created HDb (and control HWT) cells by introducing diabetic liver cytosol (or healthy liver cytosol) prepared from db/db (or wild-type) mice into rat hepatoma H4IIEC3 cells (Fig. 1b). As a result, we could successfully reproduce typical diabetic characteristics, namely a disturbed response to insulin as seen through a lack of transcriptional repression of the gluconeogenic genes PCK1 and G6PC and the aberrant secretion of glucose into the medium (Fig. 1d–f). Using the mouse liver-derived cell line, instead of rat hepatoma H4IIEC3 cells, for cell-resealing might seem to be more appropriate as mouse liver cytosol was introduced. Since our main focus was to reconstitute the perturbed response to insulin in gluconeogenesis at the cellular level, we evaluated the insulin-mediated transcriptional repression of PCK1 in human HepG2, mouse Hepa1–6, and rat H4IIEC3 cells, and chose rat H4IIEC3 cells as they showed the highest sensitivity to insulin treatment.

Interestingly, the perturbation of transcriptional control of these genes was also detected in resealed H4IIEC3 cells containing HF cytosol that had been prepared from a high-fat diet-induced obese mouse, but not in resealed cells containing ApoE cytosol that had been prepared from a mouse model of atherosclerosis, an ApoE-deficient mouse (Supplementary Fig. S2a and S2b). Thus, HDb cells showed transcriptional regulation of gluconeogenic genes that was dependent on the introduced cytosol. On the other hand, we could not detect insulin-dependent transcriptional regulation of the SREBP-1c gene in HWT and HDb cells (Supplementary Fig. S2d). As shown in Supplementary Fig. S2c, pretreatment with dexamethasone and cAMP was required for insulin-mediated transcriptional regulation of SREBP1c, suggesting that activation of several signaling molecules and specific intracellular metabolic conditions are prerequisites for the response. However, during the permeabilization process in our cell-resealing technique, these proteins and metabolites in the cytosol would flow out from the cells, which would lead to the loss of pre-activated intracellular conditions. Although dexamethasone and cAMP are also included in the cytosol and during the incubation step after resealing in the resealed cell assays, it might not be sufficient to restore insulin-mediated regulation of SREBP1c expression.

Thus, to perform cell-based assays using disease-specific model cells, it might be necessary to choose a cell line that shows the appropriate phenotype for the assay of choice.

The ratio of phosphorylated Akt to total Akt (pAkt/Akt) in cells is probably a potential biomarker for diabetic hepatic cells. We found that Akt was phosphorylated less upon insulin stimulation in HDb cells than in HWT cells (Fig. 2). This is consistent with the several reports, showing the decreased Akt phosphorylation by insulin stimulation in db/db liver or primary hepatocytes^27–29^. Given that Akt is a key enzyme for intracellular metabolism and gene transcription^25,26^, a new quantitative analytical method to evaluate the ratio of pAkt/Akt in each single cell was needed to elucidate more precisely the effect of Akt phosphorylation on the regulation of gene expression (see next paragraph).

The second aim of this study was to establish a specific analytical system to detect the various phenotypic differences between single HWT cells and single HDb cells. In particular, we focused on morphological analysis using automated quantitative image-based analysis by laser scanning confocal microscopy (LSM). Microscopic analysis seems to be particularly suitable for the phenotypic analysis of resealed cells because the introduction of fluorescently labeled cytosol into resealed cells makes them easily discernible under a fluorescence microscope. We employed the image-based quantitative analysis and PCA to characterize the diabetic phenotype of HWT and HDb cells. The quantitative data that were extracted from the images might not be totally comparable to those obtained by biochemical procedures such as WB (Figs 2 and 5). In fact, the data from the images indicated that Pio decreased the total amount of Akt in each cells, which was not detected by WB (Fig. 5b and c). On the other hand, the image-based analysis and WB yielded the same results when the cells were treated with Avs, Crz, or PF, which all substantially decreased the phosphorylation of Akt. We suppose that the image-based analysis might reveal finer distinctions than WB because the former uses data collected from single cells whereas WB shows the mean signal for all cells.

PCA is also a powerful means of identifying “feature quantities” that might reflect the differences between HWT and HDb cell populations^30–33^. Our “feature quantities” from immunofluorescence images are unique in that they contain localization and morphological information, which will be useful to eliminate data from abnormal or dead cells as they are different morphologically from normal cells. From the PCA results, we identified the ratio of pAktS473 to Akt fluorescence after cells had been treated with insulin as the index that distinguished most between HWT and HDb cells (Fig. 3c).

By screening for chemical compounds that caused an amelioration or pejoration of insulin resistance in HDb or HWT cells using the ratio of pAkt/Akt fluorescence as an index, we identified five compounds that changed the pAkt/Akt ratio from among 90 small chemical compounds of the LOPAC-Pfizer library and seven drugs used for the treatment of diabetes mellitus. Three drugs, Avs, Crz, and PF, decreased the pAkt/Akt ratio, and two drugs, Pio and Met, increased it. The decreased phosphorylation of Akt in Crz- and PF-treated cells is understandable because these compounds inhibit kinases upstream of Akt^34,35^. In contrast, no direct connection of Avs, an inhibitor of acyl-coA cholesterol acyltransferase (ACAT), to Akt phosphorylation has yet not been fully understood. It would be important to understand the mechanisms that connect ACAT activity and Akt phosphorylation because they affect not only the response of cells to insulin as shown in this study but also the aggressiveness in prostate cancer^36^.

The drugs Pio and Met, both of which are medications for the treatment of type 2 diabetes mellitus, increased the ratio of pAkt/Akt by decreasing the amount of total Akt while maintaining the amount of pAktS473 (Figs 5 and 6). Pio had a particularly marked effect on the ratio of pAkt/Akt more specifically in HDb cells than Met, which indicated that Pio might be able to ameliorate cells under diabetic conditions more effectively than Met. Pio is an agonist for PPARγ. PPARγ in liver is reportedly involved in lipid metabolism: upregulation of PPARγ contributes to hepatic steatosis^37–39^. Interestingly, hepatic PPARγ expression is elevated by 7–9 fold in ob/ob and db/db mice^40^, which suggests that Pio acts more effectively on diabetic hepatocytes than on wild-type hepatocytes. As Pio and Met, both of which are the drugs for type 2 diabetes mellitus, were extracted as compounds that could improve the diabetic phenotypes in this screening, the image-based analytical system would be valid and could be used for high-contents screening in the future study.

In conclusion, the disease-specific model cell system that can be created by using the cell-resealing technique could be a powerful platform for screening for drugs and evaluating their targets or functional mechanisms in cells under disease conditions. In addition to conventional omics data, such as that obtained by microarray analysis, proteomics, metabolomics, etc. for comparing healthy and disease states, our disease-specific model cell system coupled with a novel quantitative image-based analysis could provide morphological information such as intracellular localization or evidence about the transient accumulation of specific proteins. Such features could then become novel markers for disease state, diagnosis, and drug discovery.

## Methods

### Cells

Rat hepatoma-derived H4IIEC3 cells were purchased from ATCC. The cells were cultured in DMEM (Nissui) supplemented with 20% horse serum (ATCC), 5% fetal bovine serum (FBS; Corning), and penicillin/streptomycin (GIBCO).

### Reagents and antibodies

GTP, ATP, creatine phosphate, creatine kinase, and insulin solution (human) were obtained from Sigma. Propidium iodide (PI) and tetramethylrhodamine (TMR)-conjugated phalloidin were purchased from Molecular Probes. Dextran (10 or 40 kDa) conjugated with fluorescein or TMR was purchased from Invitrogen. Hoechst 33342 solution was obtained from Dojindo. The following primary antibodies were used: rabbit anti-phospho-Akt (S473) antibody (Cell Signaling Technology); rabbit anti-pan Akt antibody (Cell Signaling Technology); mouse anti-Akt/PKB antibody, PH Domain, clone SKB1 (Millipore, 05–591); mouse anti-GM130 antibody (BD Transduction Laboratories); rabbit anti-ERGIC53 antibody (Sigma Aldrich); rabbit anti-GRP78 BiP antibody (Abcam); mouse anti-cytochrome C antibody (BD Pharmingen); mouse anti-γ-tubulin antibody (Sigma Aldrich); mouse anti-EEA1 antibody (BD Biosciences); mouse anti-nucleoporin p62 antibody (BD Transduction Laboratories); mouse anti-β-catenin antibody (BD Transduction Laboratories); mouse anti-β-tubulin antibody (Sigma-Aldrich); Alexa 488-conjugated anti-GST antibody (Molecular Probes). The following secondary antibodies were used: Horse Radish Peroxidase (HRP)-conjugated anti-mouse IgG antibody (Promega); HRP-conjugated anti-rabbit IgG antibody (Cell Signaling); Alexa Fluor 488-conjugated anti-mouse or anti-rabbit antibodies (Life Technologies); Cy3-conjugated anti-mouse antibody (Chemicon); Alexa Fluor 546-conjugated anti-rabbit IgG antibody (Thermo Fischer Scientific); Alexa Fluor 647-conjugated anti-mouse IgG antibody (Thermo Fischer Scientific).

### Preparation of resealed cells

H4IIEC3 cells were grown on glass-bottomed dishes (Iwaki) or cover glass (Matsunami) coated with Cellmatrix Type I-C (Kurabo). The cells were washed twice with PBS and then incubated with 200 ng/ml streptolysin O (SLO; Bioacademia) on ice for 10 min. After washing three times with PBS, the cells were incubated with transport buffer (TB: 25 mM HEPES-KOH, pH 7.4, 115 mM potassium acetate, 2.5 mM MgCl_2_) at 37 °C for 5 min and then washed with TB at room temperature. These cells were called “semi-intact cells”. The semi-intact cells were incubated with cytosol (usually at a concentration of 3.0 mg/ml) and an ATP regenerating system (1 mM ATP, 50 µg/ml creatine kinase, and 2.62 mg/ml creatine phosphate), 1 mg/ml glucose, 1 mM GTP, and 100 µg/ml fluorescently labeled dextran at 37 °C for 30 min. Then 1 mM CaCl_2_ was added and the cells were incubated at 37 °C for a further 5 min. The cells were then washed twice with PBS and were further incubated with pre-warmed medium at 37 °C in 5% CO_2_ for more than 30 min.

### Preparation of cytosol

Cytosol from murine lymphoma L5178Y cells was prepared as described in Kano *et al*.^11^. Cytosol was prepared from the liver of C57BLKS/JIar-*m*+/*m*+ mice at the age of 9 weeks (Japan SLC, Inc.), C57BLKS/JIar- +*Lepr* ^db^/+*Lepr* ^db^ (db/db) mice at the age of 9 weeks (Japan SLC, Inc.), C57BL/6.KOR/StmS1c-Apoe mice at the age of 8 weeks (Japan SLC, Inc.), and C57BL/6J-DIO mice at the age of 20 weeks (Charles River) in accordance with previously described methods^3^. The experiments using mice were conducted with the approval of the animal experiment ethics committee at the Graduate School of Arts and Sciences, The University of Tokyo, and in accordance with the guidelines for the care and use of laboratory animals.

### Isolation of RNA and RT-PCR

Total RNA was purified from cells using an RNeasy Mini Kit (Qiagen) and reverse-transcribed with the use of a ReverTra Ace qPCR RT Kit (Toyobo). Real-time PCR was carried out using Fast SYBR Green Master Mix (Applied Biosystems) and a StepOnePlus Real-Time PCR System (Applied Biosystems). The primer pairs used were: forward, 5′-TGCCCATCGAAGGCATCA-3′ and reverse 5′-TCTCATGGCAGCTCCTACAAACAC-3′ for PCK1; forward, 5′-GTGGGTCCTGGACACTGACT-3′ and reverse, 5′-AATGCCTGACAAGACTCCA-3′ for G6PC; and forward, 5′-GGCAATGCTGGACCAAACAC-3′ and reverse, 5′-AAACGCTCCATGGCTTCCAC-3′ for PPIA. PPIA was used as an internal standard.

### Glucose assay

The resealed cells were incubated in medium at 37 °C for 1 hr, and then further with glucose-free medium [glucose-free DMEM without phenol red, 20 mM sodium lactate, 1 mM sodium pyruvate, 15 mM Hepes-KOH (pH 7.4)] at 37 °C for 3 hrs. The supernatant was collected and the amount of glucose was measured using a glucose assay kit (WAKO) in accordance with the manufacturer’s instructions. The values were normalized against the protein concentration in the cell lysate, as determined by a bicinchoninic acid protein assay using bovine serum albumin (BSA) as a standard.

### Immunofluorescence microscopy

Resealed H4IIEC3 cells were washed twice with PBS, fixed with 4% paraformaldehyde in PBS for 20 min, and permeabilized with 0.2% TX-100 for 15 min. The cells were blocked for 30 min in PBS that contained 3% BSA and incubated with the respective primary antibody in blocking buffer for 2 hrs at room temperature. After washing three times with PBS, the cells were incubated with the respective secondary antibody in blocking buffer for 1 hr at room temperature. After washing three times with PBS, the coverslips were mounted in SlowFade Gold antifade reagent (Invitrogen) and examined by using an LSM 710 laser scanning confocal microscope (Carl Zeiss) or an A1+ confocal laser microscope system (Nikon).

### GST-2xFYVE targeting assay

A recombinant tandem FYVE domain from mouse Hrs was produced as a GST fusion protein (GST-2xFYVE) as described in Kano *et al*.^41^. The GST-2xFYVE targeting assay was performed as described in Kano *et al*.^41^. The number of GST-2xFYVE positive dots was counted in each cell, and the means and the s.e.m. were plotted in the graph.

### Endocytosis assay for cholera toxin B subunit (CtxB)

The endocytosis assay for CtxB was performed as described in Kano *et al*.^3^ except that we used CtxB conjugated with Alexa 488, rather than Alexa 546.

### Western blotting

Western blotting was performed as described in Kano *et al*.^3^.

### Image acquisition and analysis of cells on 96-well plates

H4IIEC3 cells were grown on gelatin-coated 96-well plates (Falcon, 353219), and incubated with DMEM(-FBS) overnight. The cells were permeabilized with SLO, and WT or Db cytosol was introduced as described in “Preparation of resealed cells”. After resealing, the WT or Db cells were treated with compounds from LOPAC Pfizer (Sigma-Aldrich) at 1 or10 µM or with 1 or 10 µM pioglitazone (Toronto Research Chemicals), 0.2 or 2 mM 1,1-dimethylbiguanide (Metformin; MP Biomedicals), 20 or 200 nM glibenclamide (Wako), 10 or 100 µM nateglinide (Toronto Research Chemicals), 10 or 100 µM [D-Lys3]-GHRP-6 (Sigma), 5 or 50 mM sucralose (Sigma), or 1.8 or 18 mM saccharin (Wako) in DMEM (-FBS) at 37 °C for 1 hr. The cells were further incubated with 100 nM insulin at 37 °C for 15 or 60 min. The cells were fixed with 4% paraformaldehyde for 20 min, and subjected to immunofluorescence analysis using antibodies against pAktS473 or Akt at 4 °C overnight. After washing three times with PBS, the cells were incubated with Alexa 546-conjugated anti-rabbit IgG, Alexa 647-conjugated anti-mouse antibody, and Hoechst 33342 at room temperature for 1 hr. The immunofluorescence images were captured automatically by using an A1+ confocal laser microscope system (Nikon). Z-stack images of the cells were acquired with a slice thickness of 1 μm. NIS-Elements software (Nikon) was used for image processing at the single cell level. The areas of the nucleus, cytosol and entire cell were determined by using the binary images in the Hoechst and Alexa647(Akt) channels. The data set of the mean, sum, maximum, mean, and standard deviation of the fluorescence intensities for pAktS473 and Akt or the ratio of pAktS473 fluorescence to Akt fluorescence was measured by using NIS-Elements. The fluorescence intensities and the ratio in HWT cells treated with dmso was set to 1. The pAkt/Akt ratio in HWT cells treated with (positive) or without (negative) insulin from 4 wells were used to calculate Z’-factor, which was defined as 1-(3*SD[positive] + 3*SD[negative])/(Average[positive]-Average[negative]).

### Principal component analysis

Principal component analysis (PCA) was performed using the variables shown in Supplementary Table S1. These variables were the mean, sum, minimum, maximum and standard deviation of the fluorescence intensities of fluorescein, Akt, and pAktS473, and the area and circularity from the Cell, Nucleus or Cytosol mask. Variables that are related to fluorescence intensity indicate the amount of a given protein or other substance. All variables were calculated by using NIS-Elements at the single cell level, and normalized so that the mean was 0 and the standard deviation was 1. We then performed PCA and prepared scatter plots with all pairs of principal components for which the contribution ratio was greater than 0.028.

### Statistical analysis

Differences between individual sets of data were assessed using a Student’s t-test or a Welch’s t-test. Differences were considered significant at p < 0.05.

## Electronic supplementary material

Supplementary Information
