# Supplementary material for: Establishment and phenotyping of disease model cells created by cell-resealing technique
Source: Sci Rep. 2017 Nov 9;7:15167. doi: 10.1038/s41598-017-15443-0 (PMC5680332; doi:10.1038/s41598-017-15443-0)
Supplement: Supplementary file 1 — Supplementary Information [file 41598_2017_15443_MOESM1_ESM.pdf]

Supplementary Information

Establishment and phenotyping of disease model cells created by cell-resealing technique

Fumi Kano, Yoshiyuki Noguchi, Masayuki Murata

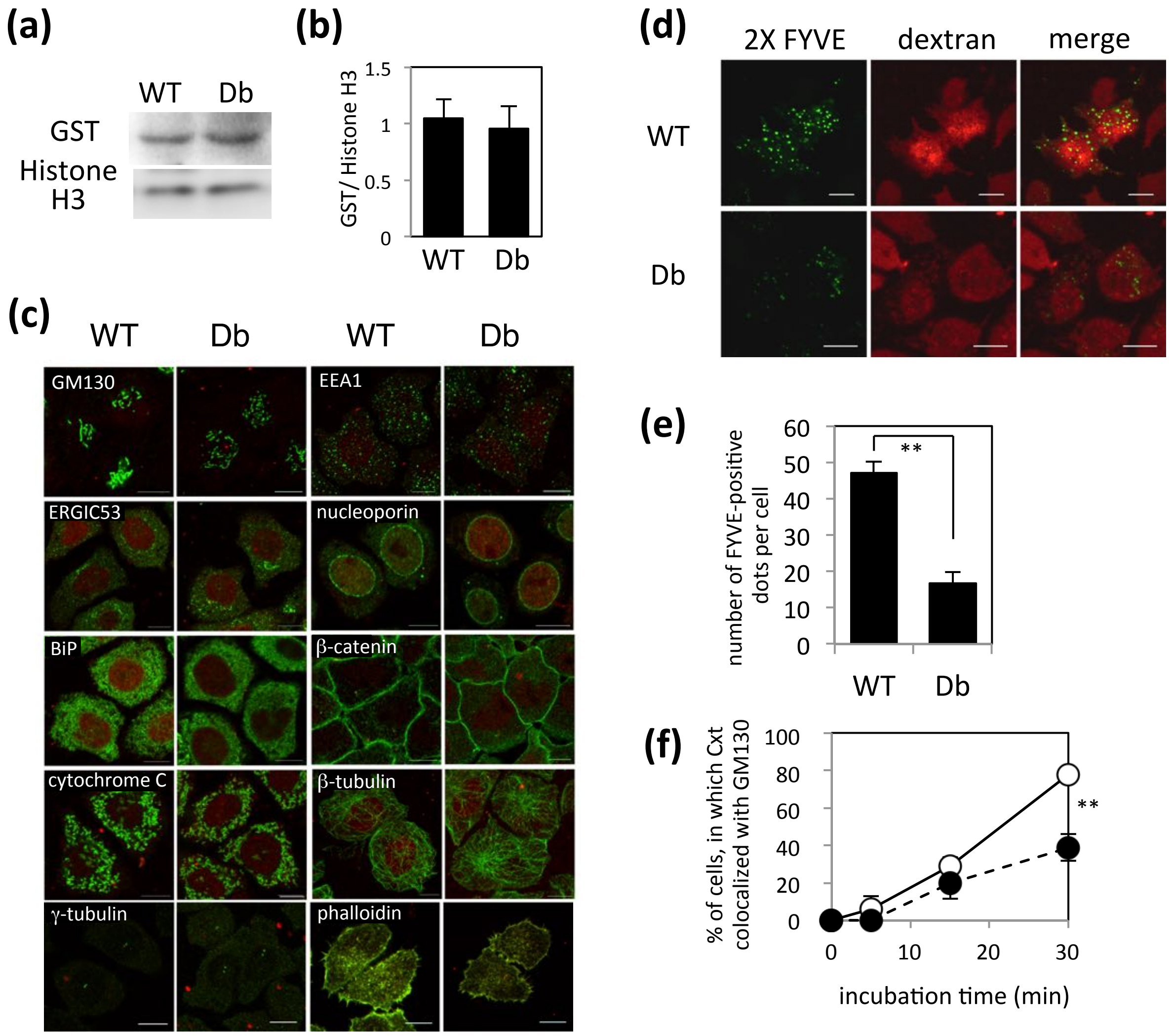

Supplementary Figure S1

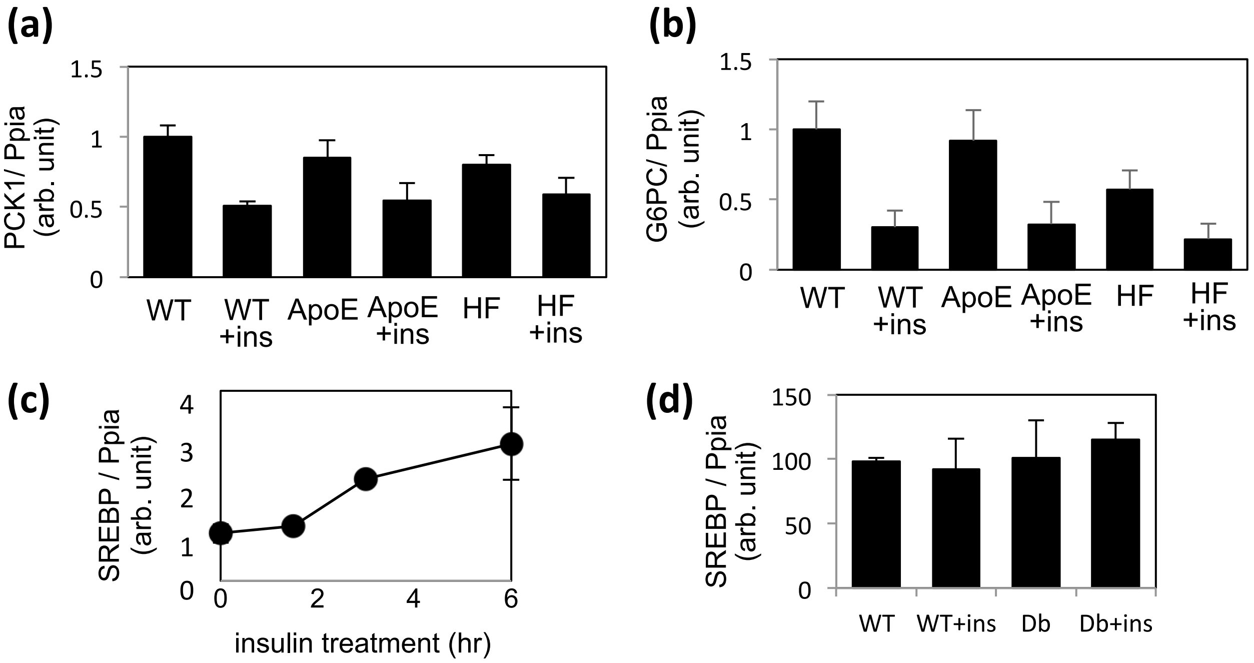

Supplementary Figure S2

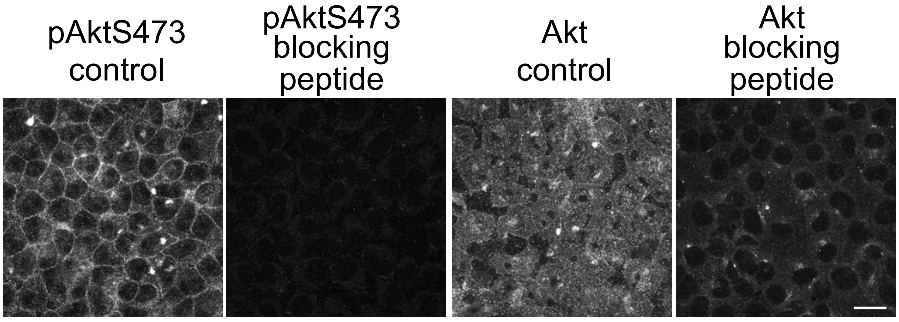

Supplementary Figure S3

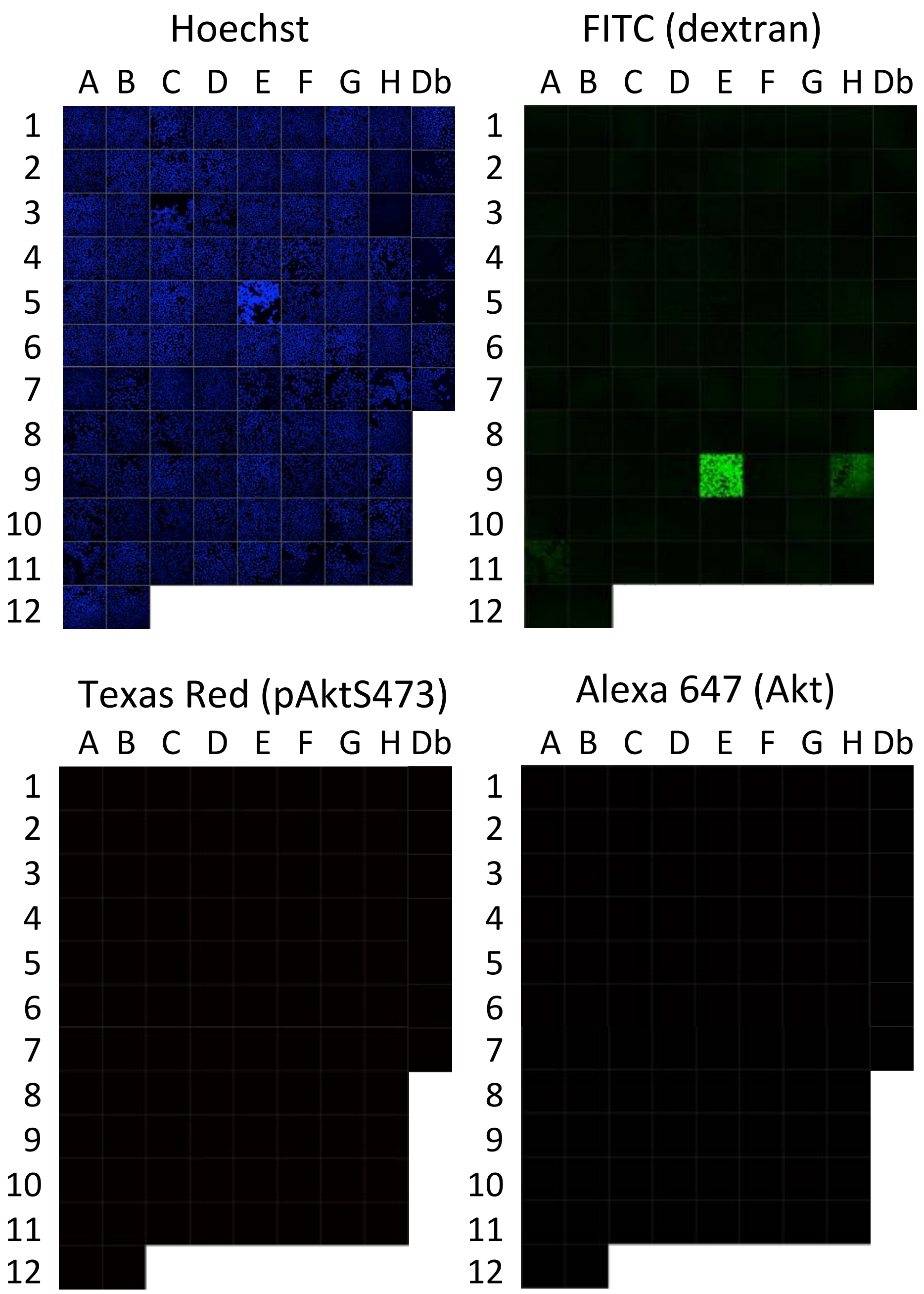

Supplementary Figure S4

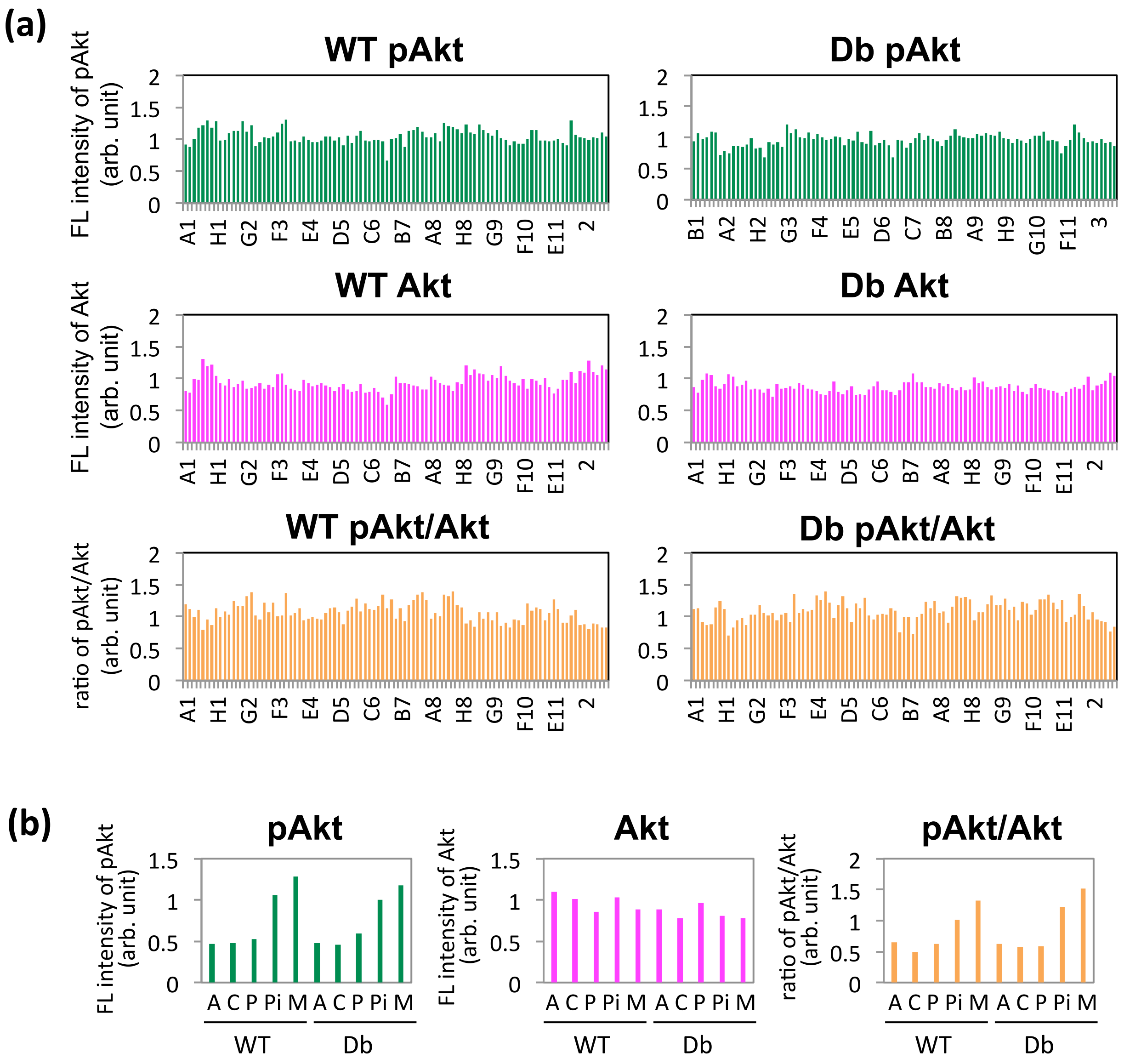

Supplementary Figure S5

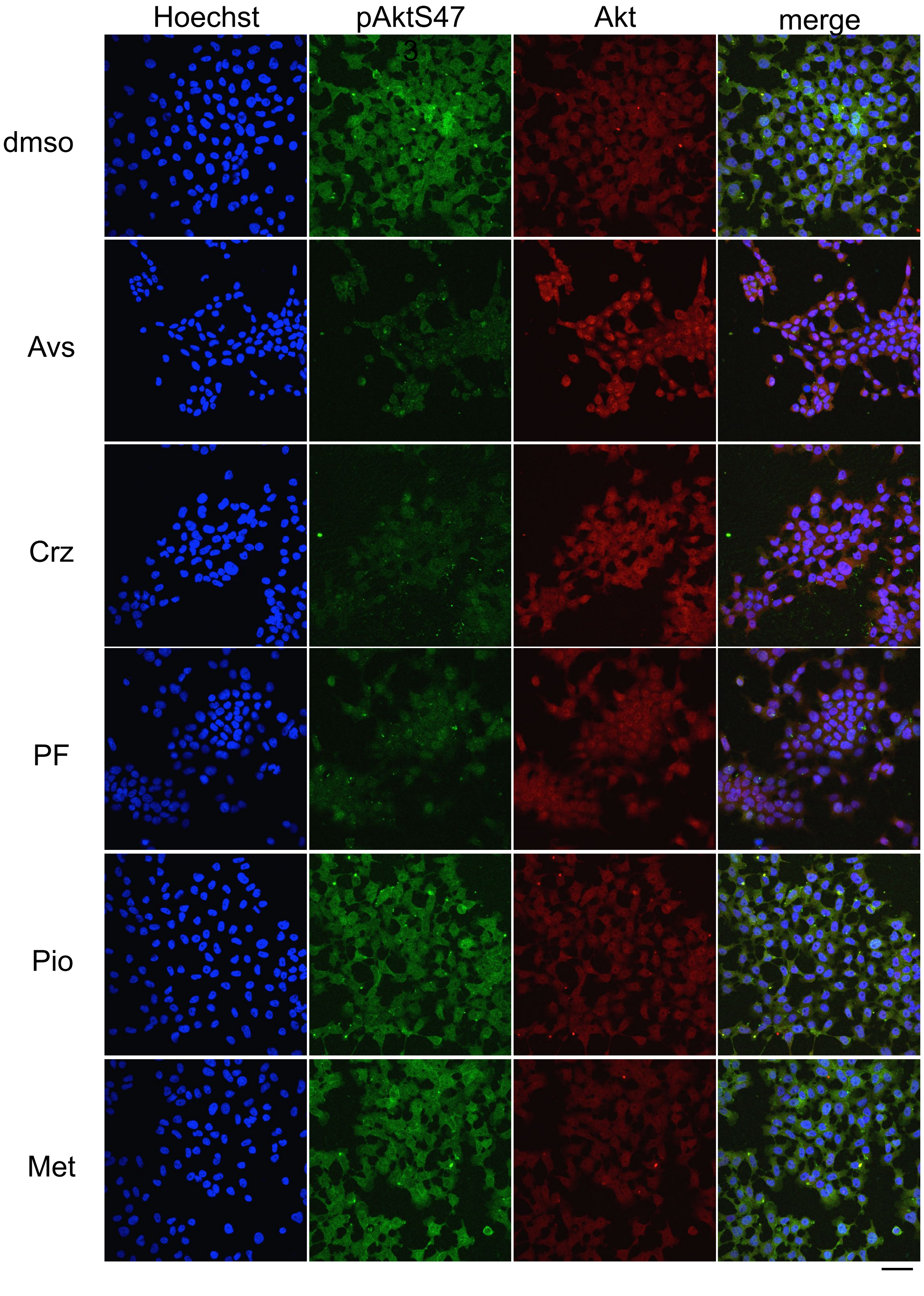

Supplementary Figure S6

Circularity of cell
Count of Akt Domain for each cell
Maximum intensity of Akt of cell
Maximum intensity of Akt(S473) of cell
Mean intensity ofAkt of cell
Mean intensity ofPAkt(S473) of cell
Minimum intensity ofAkt of cell
Minimum intensity ofPAkt(S473) of cell
Area of cell
Standard deviation of intensity of Akt of cell
Standard deviation of intensity of PAkt(S473) of cell
Sum intensity of Akt of cell
Sum intensity of PAkt(S473) of cell
Circularity of cytosol
Maximum intensity ofAkt of cytosol
Maximum intensity ofPAkt(S473) of cytosol
Mean intensity ofAkt of cytosol
Mean intensity ofPAkt(S473) of cytosol
Minimum intensity ofAkt of cytosol
Minimum intensity ofPAkt(S473) of cytosol
Area of cytosol
Standard deviation of intensity ofAkt of cytosol
Standard deviation of intensity ofPAkt(S473) of cytosol
Sum intensity of Akt of cytosol
Sum intensity of PAkt(S473) of cytosol
Circularity of nuclei
Maximum intensity ofAkt of nuclei
Maximum intensity ofPAkt(S473) of nuclei
Mean intensity ofAkt of nuclei
Mean intensity ofPAkt(S473) of nuclei
Minimum intensity ofAkt of nuclei
Minimum intensity ofPAkt(S473) of nuclei

Supplementary Table S1 Feature quantities used in PCA

	PC1	PC2	PC3	PC4	PC5	PC6	PC7	PC8	PC9	PC10	PC11
Circularity of cell	0.038306027	-0.024313476	0.091304276	0.226117676	0.318140004	0.129812391	-0.191689373	0.239921065	-0.499882607	-0.271624178	0.065190886
Count of Akt Domain for each cell	-0.227693645	-0.048012378	0.213462754	0.046955097	-0.370612348	0.162423337	-0.111110341	-0.031425712	0.239515842	-0.381165695	-0.109808766
Count of pAkt Domain for each cell	-0.305515912	-0.048802635	0.415429429	-0.063844067	-0.144207199	0.04110778	-0.00543703	0.081258271	0.075424174	-0.257886479	-0.231957111
Maximum intensity of Akt of cell	-0.409507937	-0.050728948	0.591858433	-0.09661075	-0.424856582	0.240715621	-0.093184868	0.003737122	-0.198628667	0.346062155	0.12973306
Maximum intensity of Akt(S473) of cell	-0.582357517	-0.188214342	-0.059200539	0.330872343	-0.326601906	0.211571283	-0.11074505	-0.183218525	0.226526316	-0.317589196	0.092161066
Mean intensity ofAkt of cell	-0.549057695	-0.014748313	0.641646099	-0.045427561	0.34824687	-0.198531404	0.12144204	0.107468089	0.037640044	-0.103420708	-0.132014584
Mean intensity ofPAkt(S473) of cell	-0.627901694	-0.224285062	-0.151479741	0.569141387	0.266755914	0.030807386	-0.055746022	0.175817964	-0.140451813	0.054773641	-0.073686748
Mean intensity of FITC of cell	-0.149188006	0.940261358	0.004336347	0.222598231	-0.04678428	-0.058310584	0.032222503	-0.017398608	-0.042734478	-0.011729284	0.004068813
Minimum intensity ofAkt of cell	-0.099827806	0.074993927	0.400662419	-0.062265578	0.309171451	-0.298449582	-0.227869914	0.074209528	0.245414755	-0.080136465	0.459908911
Minimum intensity ofPAkt(S473) of cell	0.012082473	0.003770403	0.120913082	0.035316188	0.193308603	-0.11039156	-0.292778898	-0.068378036	0.147914957	-0.033909684	0.727781236
Area of cell	-0.606225776	-0.000750463	-0.375775393	-0.665845129	-0.125308087	-0.056433057	-0.016287719	-0.017645028	0.029040953	0.010147303	0.045414492
Standard deviation of intensity of Akt of cell	-0.488094133	-0.047849828	0.759981078	-0.042395854	-0.202613975	0.201284887	0.005894045	-0.029199365	-0.105016291	0.116061982	0.021346739
Standard deviation of intensity of PAkt(S473) of cell	-0.672832967	-0.21858471	-0.291157634	0.515681448	-0.077362682	0.089621518	-0.003309785	-0.099101019	0.097503708	0.068189024	0.030436402
Standard deviation of intensity of FITC of cell	-0.174090392	0.935627432	0.001892089	0.21882889	-0.065923464	-0.07017934	0.04085394	-0.034699389	-0.013338249	0.010468929	0.010199274
Sum intensity of Akt of cell	-0.724284076	0.038486071	-0.110514155	-0.647660713	0.098246053	0.023887363	-0.027046767	0.070917652	0.072724813	-0.027276117	-0.03921408
Sum intensity of PAkt(S473) of cell	-0.830508784	-0.154151049	-0.379254471	-0.209811354	-0.046647561	-0.168134248	0.021779429	0.042327677	-0.081905406	0.047413084	-0.001771063
Sum intensity of FITC of cell	-0.361470552	0.802037033	-0.175858338	-0.200781606	0.112152957	0.261414446	-0.107690078	0.065990996	0.058504977	0.002851393	-0.015567996
Circularity of cytosol	0.192753301	0.039845924	0.109217524	0.051416368	0.017772364	0.189462644	0.164396471	0.023468355	0.442275833	0.179914418	-0.075446452
Maximum intensity ofAkt of cytosol	-0.382185054	-0.04043258	0.583956319	-0.087611052	-0.465405096	0.197476288	-0.167639525	0.113070581	-0.172779548	0.34745414	0.102187054
Maximum intensity ofPAkt(S473) of cytosol	-0.521758715	-0.154552624	0.049269778	0.277683982	-0.371449301	0.168450068	-0.183558158	-0.062972644	0.259673907	-0.407726104	0.041574246
Mean intensity ofAkt of cytosol	-0.465167266	-4.21E-05	0.68413966	-0.032929999	0.248838489	-0.217179562	0.013318547	0.233592578	0.051223509	-0.124253683	-0.155889101
Mean intensity ofPAkt(S473) of cytosol	-0.554030439	-0.21083694	-0.015124011	0.494829344	0.26920185	-0.015776656	-0.098826709	0.280017697	-0.13132148	-0.025569543	-0.132408687
Mean intensity of FITC of cytosol	-0.139469803	0.919608964	0.017815728	0.217356771	-0.048891193	-0.078024034	0.029788611	-0.00097077	-0.05780794	-0.025450116	-0.004542988
Minimum intensity ofAkt of cytosol	-0.04641061	0.048090127	0.157628339	-0.057927452	0.191066272	-0.249778698	-0.650381713	-0.461854614	-0.019489343	0.086911708	-0.261199082
Minimum intensity ofPAkt(S473) of cytosol	-0.00089087	0.010937054	0.059341647	-0.004622401	0.138355929	-0.193701251	-0.672684813	-0.512909928	-0.062110679	0.108138444	-0.228624663
Area of cytosol	-0.533740507	-0.088113818	-0.315285609	-0.485554677	-0.371493738	-0.415444844	0.092017929	-0.074539724	0.020365071	0.022187777	0.052120384
Standard deviation of intensity ofAkt of cytosol	-0.416149621	-0.033614312	0.751200091	-0.017072898	-0.29868794	0.16248087	-0.139673718	0.15431427	-0.084378532	0.125623904	-0.009408753
Standard deviation of intensity ofPAkt(S473) of cytosol	-0.653034561	-0.216704681	-0.111825876	0.535677981	-0.095237581	0.083978217	-0.13015986	0.078102047	0.115815205	-0.103778222	-0.048760436
Standard deviation of intensity of FITC of cytosol	-0.161677075	0.919697734	0.032811291	0.223040123	-0.061490738	-0.093408896	0.033698838	-0.01278992	-0.038823354	-0.009193607	0.002281986
Sum intensity of Akt of cytosol	-0.669134606	-0.077733199	-0.012799529	-0.463726243	-0.24833559	-0.463921125	0.08657655	0.045769395	0.042605336	-0.029561591	-0.039923612
Sum intensity of PAkt(S473) of cytosol	-0.74899478	-0.174359779	-0.299094835	-0.216269821	-0.210130748	-0.372154797	0.040186971	0.074384237	-0.042562242	0.015584032	-0.03491009
Sum intensity of FITC of cytosol	-0.331703454	0.819120975	-0.105331146	0.033758047	-0.171876213	-0.203519945	0.056566915	-0.015358702	-0.039843962	-0.004344666	0.006821506
Circularity of nuclei	-0.180074772	-0.062522665	-0.159120181	-0.080763926	-0.098535673	-0.156035238	-0.149632636	0.097689578	-0.572587873	-0.325812977	0.187216494
Maximum intensity ofAkt of nuclei	-0.428583784	-0.059578379	0.373953017	-0.078312114	0.232172534	0.136357904	0.378698819	-0.488806715	-0.129176197	-0.066021675	0.109532425
Maximum intensity ofPAkt(S473) of nuclei	-0.664342617	-0.228431282	-0.27952232	0.416908539	0.040319646	0.08485872	0.101963919	-0.212675291	-0.023652845	0.098116278	0.081943504
Mean intensity ofAkt of nuclei	-0.607210371	-0.043808475	0.441332297	-0.040143524	0.424689588	-0.175095394	0.253684086	-0.093367359	0.027553069	-0.040202504	-0.063578679
Mean intensity ofPAkt(S473) of nuclei	-0.686683137	-0.226592447	-0.315662336	0.508660444	0.100658772	-0.04380208	0.015182932	0.036410969	-0.037383775	0.165464821	-0.018956303
Mean intensity of FITC of nuclei	-0.170417702	0.937855538	-0.016705753	0.22150422	-0.076946803	-0.077278394	0.040466606	-0.030037995	-0.021323616	0.003628423	0.008226449
Minimum intensity ofAkt of nuclei	-0.26085787	0.036226535	0.319018831	0.049779467	0.2829971	-0.337856471	-0.098358083	0.240962969	0.329237803	0.121687673	0.013472603
Minimum intensity ofPAkt(S473) of nuclei	-0.261789336	-0.119279474	-0.145319019	0.339023794	0.125049647	-0.103002109	-0.135665615	0.261290162	0.200824538	0.288385837	0.024028946
Area of nuclei	-0.425557284	0.118564182	-0.284973958	-0.604507158	0.267687887	0.458465379	-0.156268971	0.067964104	0.0274721	-0.010925397	0.01535352
Standard deviation of intensity ofAkt of nuclei	-0.445539338	-0.04683202	0.435935302	-0.053701161	0.316400005	0.095085503	0.408757557	-0.464899701	-0.085425159	-0.062211125	0.067595517
Standard deviation of intensity ofPAkt(S473) of nuclei	-0.582194939	-0.209014477	-0.315676695	0.438625111	0.041716111	0.104159192	0.125191872	-0.220475598	0.016467329	0.170578471	0.079748599
Standard deviation of intensity of FITC of nuclei	-0.171454492	0.887677206	-0.002377552	0.209018535	-0.048727109	-0.04475267	0.049089252	-0.043962	0.013331446	0.02034219	0.008863853
Sum intensity of Akt of nuclei	-0.487404069	0.131124762	-0.157314039	-0.560878798	0.381080346	0.467475232	-0.121824571	0.066066547	0.071772263	-0.014303465	-0.022956547
Sum intensity of PAkt(S473) of nuclei	-0.778155843	-0.103331327	-0.40528642	-0.165121885	0.151766282	0.098955679	-0.003489688	-0.002457735	-0.113152381	0.076032236	0.037086929
Sum intensity of FITC of nuclei	-0.321975614	0.679172306	-0.180396708	-0.271238349	0.216361228	0.420775543	-0.161780158	0.090954967	0.091752737	0.005490145	-0.022812732

Supplementary Table S2

	PC1	PC2	PC3	PC4	PC5	PC6	PC7
Circularity of cell	0.120116997	0.149144775	0.36642721	-0.136117119	-0.209916438	-0.439368922	0.415177304
Count of Akt Domain for each cell	0.144899476	-0.252341458	0.143698319	0.550398897	0.077390957	-0.223381066	-0.112365091
Maximum intensity of Akt of cell	0.378490099	-0.586726673	0.183511841	0.556627124	0.124934532	-0.167940218	-0.052180288
Maximum intensity of Akt(S473) of cell	0.780886394	0.386537039	0.213627299	0.062551779	-0.022524234	0.057993959	-0.044632092
Mean intensity ofAkt of cell	0.422246976	-0.795789399	0.232035216	-0.231291544	-0.070230731	0.163995173	0.066579073
Mean intensity ofPAkt(S473) of cell	0.811802159	0.440624107	0.28292696	-0.029503137	-0.089753605	0.01604743	-0.029205898
Minimum intensity ofAkt of cell	0.254171551	-0.620185632	0.22999681	-0.439039506	0.093797569	0.109283825	-0.095576527
Minimum intensity ofPAkt(S473) of cell	0.277945224	0.06710251	0.202344073	-0.231764141	0.037124275	-0.140787552	-0.321103659
Area of cell	0.593789681	-0.059772788	-0.779756767	0.028162797	0.090226276	-0.068299441	0.026792901
Standard deviation of intensity of Akt of cell	0.418146089	-0.75381943	0.260546693	0.351274114	0.005815014	-0.036133479	0.011868791
Standard deviation of intensity of PAkt(S473) of cell	0.787101067	0.493802861	0.242543605	0.053468076	-0.028470681	0.083066673	-0.030501126
Sum intensity of Akt of cell	0.679600882	-0.449555091	-0.542020871	-0.092071627	0.016703628	0.010578665	0.0423551
Sum intensity of PAkt(S473) of cell	0.856089518	0.193791793	-0.438600042	0.016100557	0.051164631	-0.011677671	0.02672846
Circularity of cytosol	-0.437775892	-0.103240778	0.004627084	0.156776765	-0.200303393	0.390971548	-0.222200738
Maximum intensity ofAkt of cytosol	0.34630084	-0.583466275	0.220625472	0.525946392	0.231795508	-0.138551715	-0.081683302
Maximum intensity ofPAkt(S473) of cytosol	0.768275353	0.365503946	0.207538717	0.055469074	0.0114842	0.065069461	-0.045051989
Mean intensity ofAkt of cytosol	0.361932973	-0.792320271	0.279737875	-0.206232261	0.019014996	0.174696865	0.035843661
Mean intensity ofPAkt(S473) of cytosol	0.787447168	0.377733068	0.309425641	-0.051433288	-0.091613599	0.027886891	-0.049646664
Minimum intensity ofAkt of cytosol	0.187510497	-0.443577094	0.179920984	-0.469452307	0.302695761	-0.329267722	-0.161838611
Minimum intensity ofPAkt(S473) of cytosol	0.135801272	-0.019590984	0.13922193	-0.340925174	0.314275608	-0.616166903	-0.267851564
Area of cytosol	0.602857959	0.099972736	-0.597154021	0.029705627	0.461450705	0.09568269	0.117753397
Standard deviation of intensity ofAkt of cytosol	0.341706479	-0.71493187	0.309291579	0.389002177	0.138743974	-0.045256432	-0.046613958
Standard deviation of intensity ofPAkt(S473) of cytosol	0.789830447	0.475492617	0.251165144	0.046716399	-0.036946922	0.064878831	-0.033493358
Sum intensity of Akt of cytosol	0.683851962	-0.254959941	-0.453410086	-0.062482113	0.422760239	0.152165955	0.116541265
Sum intensity of PAkt(S473) of cytosol	0.762269328	0.227016232	-0.402506822	0.023153719	0.365477321	0.119969755	0.081450058
Circularity of nuclei	0.087332525	0.169141127	0.282174012	-0.055119951	0.093449517	-0.196930167	0.703304391
Maximum intensity ofAkt of nuclei	0.455285244	-0.639276461	0.054082959	0.069369814	-0.268910049	0.020900442	0.133795717
Maximum intensity ofPAkt(S473) of nuclei	0.822214573	0.441595497	0.204561311	0.015410971	-0.105101301	0.02207047	-0.019454321
Mean intensity ofAkt of nuclei	0.47920484	-0.742653659	0.167959247	-0.245098797	-0.11906837	0.16621823	0.100207319
Mean intensity ofPAkt(S473) of nuclei	0.804301657	0.482078776	0.237402945	-0.001967592	-0.063721219	0.032939353	-0.017640393
Minimum intensity ofAkt of nuclei	0.31322263	-0.568672483	0.2396551	-0.416619653	0.093601063	0.15887876	-0.071060366
Minimum intensity ofPAkt(S473) of nuclei	0.510353798	0.384408483	0.199655578	-0.048147056	-0.020822908	0.032685525	-0.170418205
Area of nuclei	0.302162503	-0.260769084	-0.675686371	0.012735801	-0.484373432	-0.27136922	-0.116198604
Standard deviation of intensity ofAkt of nuclei	0.452772314	-0.695106579	0.128103	0.009629082	-0.227489992	0.097407901	0.139638434
Standard deviation of intensity ofPAkt(S473) of nuclei	0.76290087	0.460437676	0.234544581	0.024315133	-0.067365332	0.053121563	-0.025029449
Sum intensity of Akt of nuclei	0.461782831	-0.528502313	-0.472242796	-0.096643223	-0.447300673	-0.152704516	-0.054737359
Sum intensity of PAkt(S473) of nuclei	0.750491687	0.091127369	-0.367183541	0.001362989	-0.417796364	-0.198767493	-0.05993978

Supplementary Table S3

Supplementary Figure Legends

Supplementary Figure S1 Comparison of the inflow of added protein, morphology of intracellular structures, and Ctx transport between HWT and HDb cells.

(a) Semi-intact H4IIEC3 cells were incubated with WT or Db cytosol in the presence of 10 μg recombinant GST protein for 30 min. After resealing, the cells were incubated with medium for 1 hr, lysed, and were subjected to Western blotting using anti-GST or anti-Histone H3 antibody.

(b) The signals corresponding to GST and Histone H3 were quantified, and the mean and the s.e.m. from three independent experiments are shown in the graph.

(c) H4IIEC3 cells were permeabilized with SLO, and incubated with WT or Db liver cytosol that contained TMR-dextran (red). After resealing, the cells were subjected to immunofluorescence using antibodies against GM130, ERGIC53, BiP, cytochrome C, γ-tubulin, EEA1, nucleoporin, β-catenin, β-tubulin, or fluorescently labelled phalloidin (green). Bar = 10 μm.

(d) Visualization of intracellular PI3P in HWT and HDb cells. The cells were permeabilized, and incubated with WT or Db liver cytosol that contained GST-2xFYVE recombinant protein (2xFYVE) and TMR-dextran (dextran). After resealing, the cells were stained with an Alexa 488-conjugated anti-GST antibody. Bar = 10 μm.

(e) The cells were treated as described in (d), and the number of GST-2xFYVE positive dots per cell was counted, and the means and the s.e.m. from five independent experiments are shown in the graph. **p = 0.0001175.

(f) The number of cells in which Cholera toxin B subunit (CtxB) was accumulated at the Golgi on the basis of the colocalization of CtxB with GM130 was counted after a 0, 5, 15, and 30 min chase in WT (○) and Db (●) cells. The means and standard deviations for the percentages of these cells are shown in the graph. Three independent experiments were performed. **p=0.00032 at 30 min.

Supplementary Figure S2 PCK1 and G6PC expression in WT, ApoE, and HF model cells, and SREBP1 expression in WT and Db cells

(a) and (b) Serum-starved H4IIEC3 cells were permeabilized with SLO and then incubated with liver cytosol prepared from WT, ApoE, or high-fat diet-induced obese (HF) mice at 37°C for 30 min. After resealing and incubation with DMEM(-FBS) for 1 hr, the cells were further incubated in the presence or absence of insulin for 1 hr. The relative expression levels of PCK1 (a) and G6PC (b) were obtained by RT-PCR. The levels of expression in WT cells without insulin treatment were set to 1. The means and standard deviations from three independent experiments are shown in the graph.

(c) H4IIEC3 cells were pretreated with 10 μM cAMP and 250 nM dexamethazone in DMEM (-FBS) overnight, and then treated with 100 nM insulin for 0, 1.5, 3, or 6 hrs. The relative expression level of SREBP was examined by RT-PCR. The expression level in cells without insulin treatment was set to 1. The means and standard deviations from three independent experiments are shown in the graph.

(d) H4IIEC3 cells were pretreated as described in c, permeabilized with SLO, and incubated with WT or Db liver cytosol. After resealing and incubation with DMEM (-FBS), the cells were treated with or without insulin for 3 hrs. The relative expression level of SREBP was examined by RT-PCR. The expression level in WT cells without insulin treatment was set to 100 %. The means and standard deviations from three independent experiments are shown in the graph.

Supplementary Figure S3 An immunizing peptide blocking experiment for immunofluorescence staining of pAktS473 and Akt

Anti-pAktS473 or anti-Akt antibodies were preincubated in the presence or absence of the immunized peptide (2x volume of Phospho-Akt (Ser473) Blocking Peptide [CST, #1140] to anti-pAktS473 antibody and 5x amount of PKB alpha [GST-Tagged] [Ubiquigent, 66-0018-050] to anti-Akt antibody) at r.t. for 2 hrs. Serum-starved H4IIEC3 cells were treated with 100 nM insulin for 15 min, and fixed with 4% PFA for 20 min. The cells were subjected to the immunofluorescence analysis using anti-pAktS743 or anti-Akt antibodies preincubated with (blocking peptide) or without

(control) immunized peptide. Bar = 20 μm.

Supplementary Figure S4 Fluorescent images of intact H4IIEC3 cells that had been treated with a library of small chemical compounds, at the wavelength used for imaging pAktS473, Akt, and fluorescein-dextran.
H4IIEC3 cells that had been grown on gelatin-coated 96 well plates were incubated with medium lacking FBS overnight. After treatment with 90 LOPAC library drugs and 7 of diabetic drugs (Db) for 1 hr, the cells were further incubated with 100 nM insulin for 1 hr. The cells were fixed and stained with Hoechst33342. The fluorescent images of Hoechst33342, FITC (dextran), TexasRed (pAktS473), and Alexa647 (Akt) were obtained by NIKON A1 confocal microscopy equipped with an automatic image acquisition system.

Supplementary Figure S5 Quantification of the mean fluorescence intensity of pAktS473 and Akt, and the ratio of pAktS473 fluorescence to Akt fluorescence in HWT and HDb cells treated with a library of small chemical compounds at different concentrations of drugs.
(a) HWT or HDb cells on 96-well plates were incubated with small chemical compounds from LOPAC-Pfizer at 1 μM or the seven additional drugs (the working concentrations are 10-fold diluted to the original one) for 60 min and then 100 nM insulin was added for 60 min. The cells were further subjected to immunofluorescence using anti-pAktS473 and anti-Akt antibodies and the images were obtained using the automated image acquisition system. The mean fluorescence intensities of pAktS473 and Akt, and the mean ratio of pAktS473 fluorescence to Akt fluorescence are shown in the graph.
(b) HWT and HDb cells were incubated with Avs (A), Crz (C), PF (P), and Pio (Pi) at 5 μM and Met (M) at 1 mM (2-fold diluted concentration compared to Fig. 4) for 1 hr and further with insulin for 1hr. The cells were stained with anti-pAktS473 and anti-Akt antibodies and the fluorescent images were analyzed as described above. The mean fluorescence intensities of pAktS473 and Akt, and the mean ratio of pAktS473 fluorescence to Akt fluorescence are shown in the graph.

Supplementary Figure S6 Immunofluorescence images of H4IIEC3 cells treated with DMSO and each of the five drugs after insulin treatment and staining with anti-pAktS473 and Akt antibodies.
H4IIEC3 cells were incubated with DMSO, Avs, Crz, PF, Pio, or Met for 1 hr and then with insulin for a further 1 hr. The cells were fixed and stained with antibodies against pAktS473 (green) and Akt (red) and with Hoechst 33342 (blue). Bar = 50 μm.

Supplementary Table S1 Feature quantities used in PCA

Supplementary Table S2 Loadings for PCA in Figure 3C

Supplementary Table S3 Loadings for PCA in Figure 6B

Supplementary Methods

Lactate dehydrogenase (LDH) assay

H4IIEC3 cells were treated with or without SLO on ice, washed with ice-cold PBS three times, and incubated with TB at 37°C for 5 min. The supernatant was collected (sup1). Then the cells were incubated with TB at 37°C for 30 min and the supernatant was collected (sup2). The cells were scraped, collected, and passed through a 27 gauge needle 30 times. After centrifugation at 1,000g at 4°C for 5 min to remove nuclei, the supernatant was centrifuged at 65,000 rpm at 4°C for 60 min in Optima TLX ultracentrifuge (Beckman Coulter) using a TLA-110 rotor, and the supernatant was collected (cytosol). The LDH activity in sup1, sup2, and cytsol was determined using cytotoxicity LDH assay kit-WST (Dojindo) according to the manufacturer's instruction. The cytosol leakage was estimated as (sup1+sup2)/(sup1+sup2+cytosol).

Preparation of recombinant GST protein and Western blotting

Recombinant GST protein was produced as described in Matsuto et al.[14]. Western blotting was performed using mouse anti-GST antibody (Cell Signaling Technologies, #2624) and rabbit anti-Histone H3 antibody (Cell Signaling Technologies, #4499).

Figure 2a

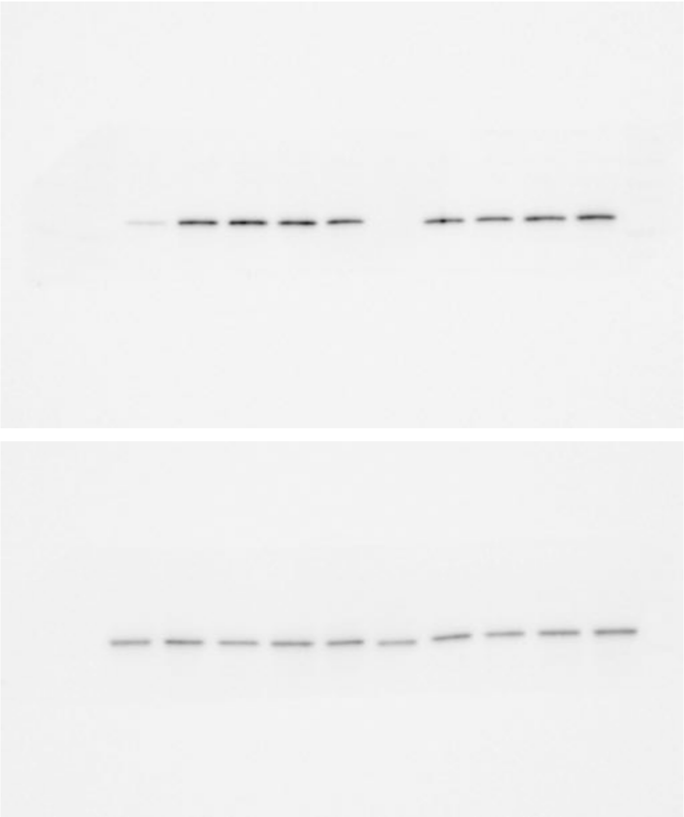

pAktS473

pAkt

Figure 5c

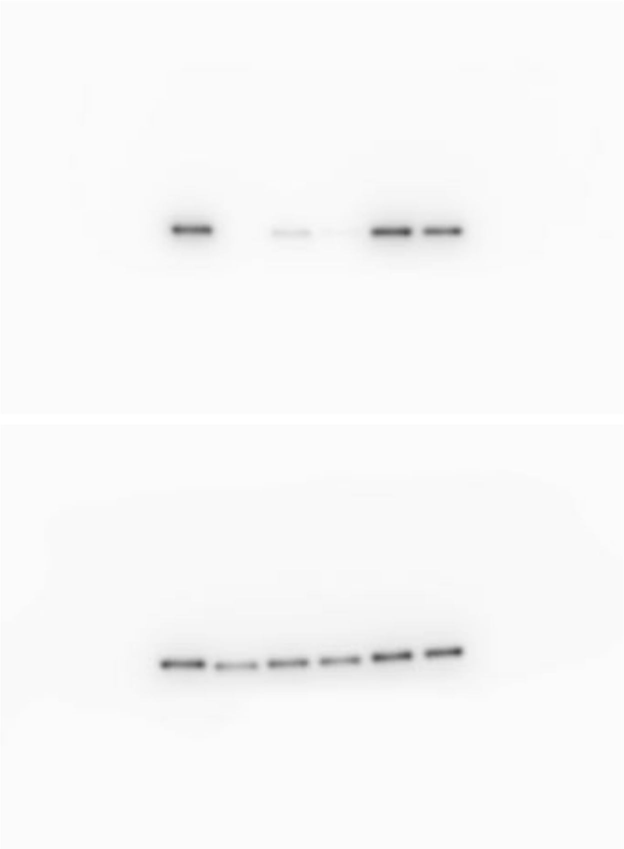

pAktS473

pAkt

Supplementary Information
